# Phenolic Compound Induction in Plant-Microbe and Plant-Insect Interactions: A Meta-Analysis

**DOI:** 10.3389/fpls.2020.580753

**Published:** 2020-12-15

**Authors:** Christopher M. Wallis, Erin R.-A. Galarneau

**Affiliations:** ^1^Crop Diseases Pest and Genetics Research Unit, USDA-ARS San Joaquin Valley Agricultural Sciences Center, Parlier, CA, United States; ^2^Viticulture and Enology Department, University of California, Davis, Davis, CA, United States

**Keywords:** plant defense responses, secondary metabolism, phenolics, plant breeding, systemic acquired resistance, induced systemic resistance

## Abstract

Plants rely on a variety of ways to protect themselves from being fed upon, including *de novo* production of specific compounds such as those termed as phenolics. Phenolics are often described as important in plant health and numerous studies have concluded they increase as a result of insect feeding, pathogen infection, or beneficial microorganism colonization. However, there are some studies reaching differing conclusions. Therefore, meta-analyses were conducted to observe whether common trends in phenolic induction in plants can be made when they become hosts to insects or microorganisms. Four hypotheses were tested. The first was that total phenolics increase as a generic response, and meta-analyses confirmed that this occurs when plants are infested with insects or colonized by bacterial or fungal microorganisms, but not for oomycetes. The second hypothesis was that phenolic induction is different when a beneficial microorganism colonizes a plant vs. when a plant is infected by a pathogen. Beneficial bacteria, pathogenic bacteria, and beneficial fungi produced increased phenolic levels in plant hosts, but fungal pathogens did not. The third hypothesis was that insect feeding method on plant hosts determines if phenolics are induced. Chewing induced phenolics but piercing-sucking and wood-boring did not. Lastly, we used meta-analyses to determine if annual or perennials rely on phenolic induction in different amounts, and even though annuals had significantly increased phenolic levels but perennials did not, it was observed that phenolic induction was not statistically different when plant type was considered. These results demonstrate that phenolic induction is a common response in plant hosts exposed to feeding or colonization, with specific exceptions such a pathogenic fungi and piercing-sucking insects.

## Introduction

**Figure 1:**
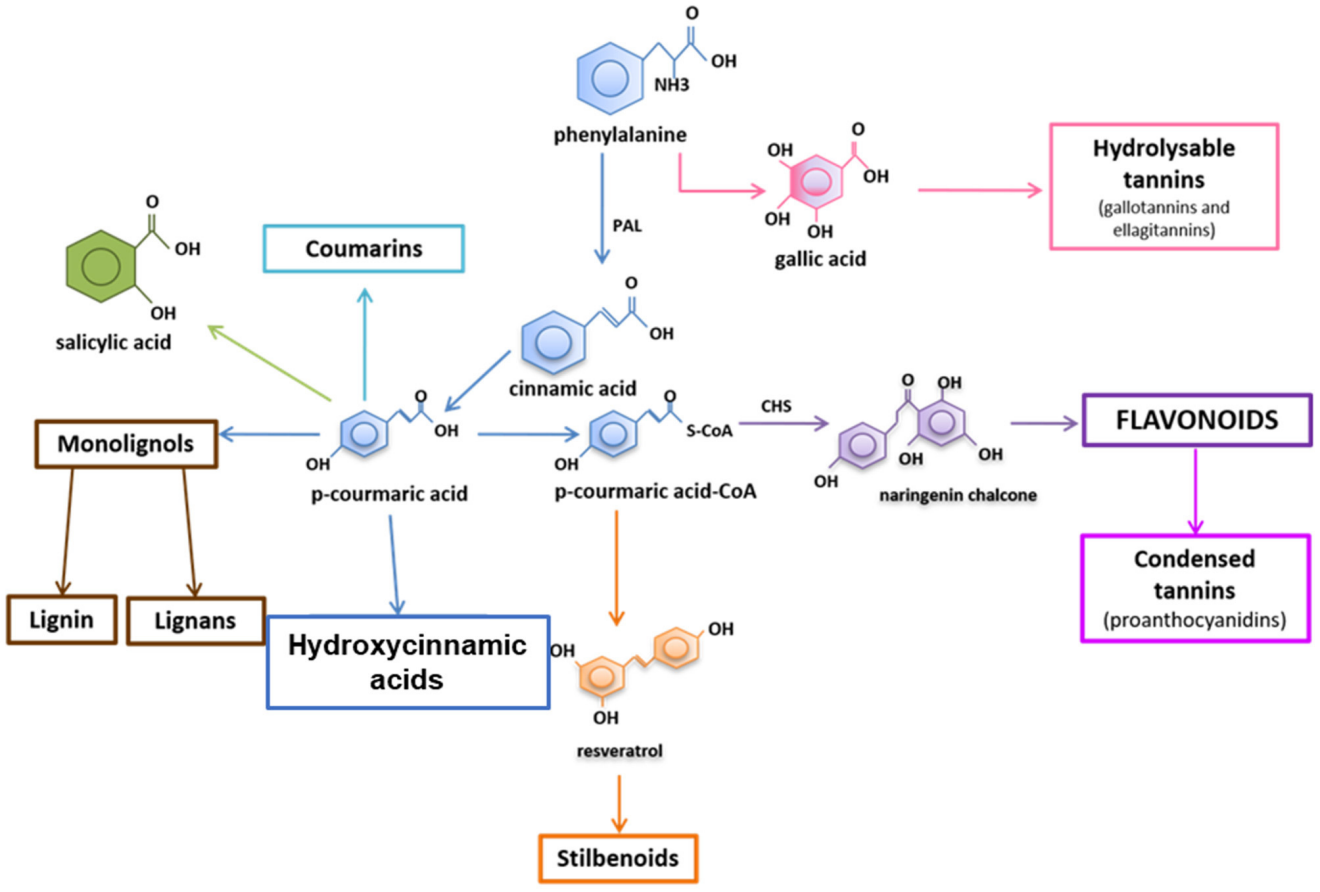
Simplified overview of phenolic pathways. The major subclasses of phenolics include the phenolic acid in blue and flavonoids in purple (including condensed tannins). Hydroxycinnamic acids, or HCAs, are comprised mostly of phenolic acids but could also include monolignols, coumarins, and other downstream compounds except the flavonoids and stilbenoids.

Plants possess a wide variety of compounds to assist in phenotypic plasticity, as escape from stressors is not possible (Frankel, 1959; Paul et al., 2000; Stout et al., 2006; Kaplan et al., 2008b). Among these compounds is a class termed phenolics which contains phenolic acids (often termed hydroxycinnamic acid derivatives or HCAs), flavonoids, polyphenols, stilbenoids, and more (Figure 1). Phenolics are defined as compounds with at least one phenyl ring. Phenolic compound functions are diverse in plants, ranging from roles in growth and development (e.g., cell wall thickening, hormone production, and pigmentation), reproduction (e.g., pigmentation, fruit flavoring, and fruit protection), and defense against stressors (e.g., osmoregulation, UV protection, anti-herbivory roles, and antimicrobial activity) (Dixon, 2001). Historically, studies examining non-cell wall associated phenolic compounds during plant defense have focused on total phenolic levels or levels of two major subclasses of phenolics: flavonoids or hydroxycinnamic acids (HCAs) (Figure 1). This is likely due to the logistics of examining phenolics from plant tissues, which to gain data on individual compounds involves chromatography equipment and expertise, whereas fast colorimetric techniques exist for quantifying total phenolics, total flavonoids, or total HCAs.

However, an attempt to quantify trends that may exist across all these studies to generalized hypotheses has not been done, especially drawing in both insects and microorganisms as the inducers of physiological changes within plants. Indeed, very few studies to date examined effects of both insects and microorganisms on plant physiology, despite both are widely expected to colonize plants in tandem or sequentially (Tack and Dicke, 2013). Therefore, we conducted multiple meta-analyses using studies from 2008 to 2017 to explore common trends in induced phenolic production. Moderator analyses were then conducted to address additional specific hypotheses about the reliance that plants may have on phenolics to counter insects or pathogens. Further, moderator analyses also assessed phenolic induction upon beneficial microorganism colonization of plants. All of the hypotheses were tested using metadata of total plant phenolic levels. In addition, total plant flavonoid or HCA levels were assessed separately when enough observations in the papers were available. These subdivisions were assessed separately because of differences in the methodology to calculate levels (i.e., various adjustments in colorimetric techniques) as well as potential differences in co-regulation via plant responses (Wallis et al., 2008).

Any form of wounding or damage, biologically induced, generates numerous responses for “healing” or tissue repair, which includes the delayed response of induced phenolics (Bostock and Stermer, 1989; Paul et al., 2000). Current research of the role of phenolics in plant interactions with other organisms has primarily focused on determining their role in protection during attack by insects and pathogens (bacteria, fungi, nematodes, and viruses) (Hammerschmidt, 1999; Simon and Hilker, 2003; Stout et al., 2006). Overall, during plant -organism interactions, phenolics are believed to increase due to any form of damage caused by the insect pests or microbes (Nicholson and Hammerschmidt, 1992; Stout et al., 2006), but this trend has not been formally analyzed across plants and attack organisms. Thus, our overall meta-analysis was to examine a hypothesis that plants, as a general defense response, will have significantly greater phenolic levels in plants fed upon by insects or colonized by microorganisms as compared to untouched plants.

Although the general response is expected that phenolics increase due to insect/microorganism presence, our second hypothesis was that plants would produce greater phenolics when infected with a beneficial vs. a pathogenic organism. A “beneficial” is defined here as an organism that, when inoculated to the plant does not harm the plant and can impact subsequent infection by a pathogen or otherwise promote plant health. “Pathogen” is defined as an organism that causes damage and reduces the productivity of a plant (e.g., yield, photosynthesis). Most studies investigate bacterial and fungal beneficials, followed by a bacterial or fungal pathogen infection, and thus our meta-analysis focused on these experiments. The role pathogenic or beneficial organisms may have in priming plant defenses was previously assumed (Van der Ent et al., 2009; Gamir et al., 2014; Wilkinson et al., 2019; Djellout et al., 2020), including inducing phenolics (Schulz et al., 1999; Panina et al., 2007; Shoresh et al., 2010). In addition, pathogens can actively use suppressors and enzymes to mitigate phenolic production to some degree (Vidhyasekaran et al., 1992; Lyngkjaer and Carver, 2000; Shalaby and Howitz, 2015; Mason et al., 2016).

Considering that phenolics can be induced by wounding, a more systematic look was taken with insect feeding styles, with our third hypothesis anticipating that insects that macerate (chew) plant tissue would trigger phenolics in greater concentrations than those that feed by piercing-sucking, as the latter are hypothesized or shown previously to cause reduced damage or actively suppress defense responses (Zarate et al., 2007; Rodriguez-Saona et al., 2010; Alba et al., 2011; Bidart-Bouzat and Kliebenstein, 2011; Ali and Agrawal, 2012). In addition, wood-boring insects were considered separately from the two above groups and anticipating being between the two groups due to dwelling within the plant tissue but still cause feeding damage, resulting in differences in host physiological responses (Wallin and Raffa, 2000; Franceschi et al., 2005; Erbilgin et al., 2006).

Lastly, we considered the lifestyle of the plant itself, considering if the plant was grown as an annual (primarily vegetative green tissue that would die or be removed at the end of a growing season) or a perennial (often developing wood and persisting year-to-year). The hypothesis being that perennials might rely greater on host phenolic production in response to insect feeding or microorganism colonization than herbaceous annual plants because the plant must persist longer before reproduction (Massad, 2013). This is consistent with implications of the Optimal Defense, Plant-Age, Carbon-Nutrient Balance, and Growth-Differentiation Balance hypotheses (Feeny, 1976; Herms and Mattson, 1992; Barto and Cipollini, 2005; Massad et al., 2012, 2014). In other words, growth and reproduction may be more important for annual plants than investing resources into compounds for defense when the plant only has one season to spread its progeny (Leimu and Koricheva, 2006).

Investigating these hypotheses will improve understanding about how plants, in general, rely on phenolic compounds for improved health and protection. These results will also provide some insights about defense responses across a wide variety of plant under attack by insects or microorganisms, or colonized by microorganisms. This is considered of great importance to gain a complete understanding of plant-microbe and plant-insect interactions (Beckers and Spoel, 2006; Thaler et al., 2012).

## Materials and Methods

**Figure 2:**
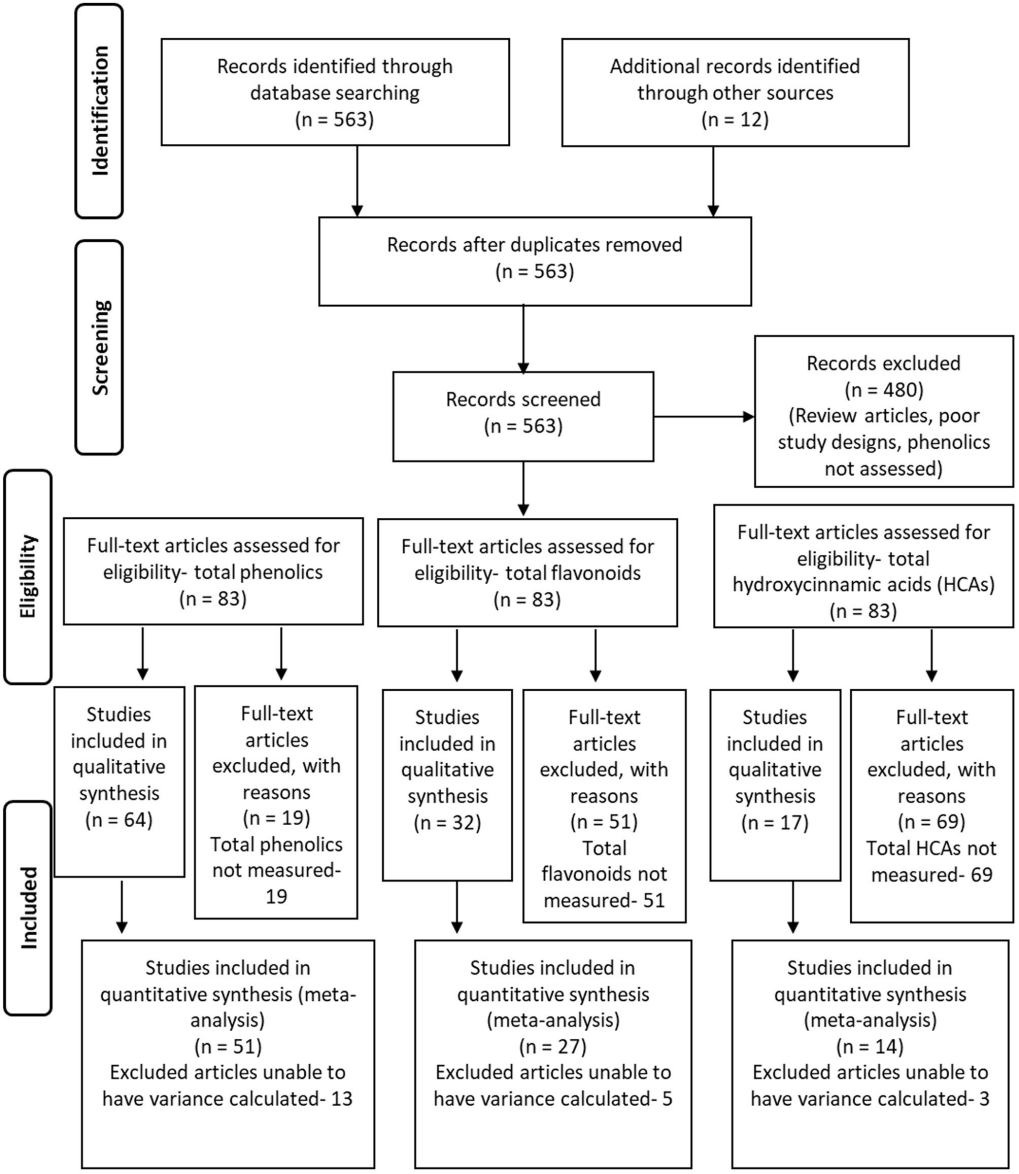
The PRISMA flowchart summarizing the selection of studies for use in the different meta-analyses performed, including those on total phenolic levels, total flavonoid levels, and total hydroxycinnamic acid levels. Adapted from Moher et al. (2009).

A PRISMA workflow diagram for article identification, screening, determination of eligibility, and determination of inclusion for the meta analyses is provided in Figure 2 (Moher et al., 2009). A total of 563 articles found by Web of Science (http://apps.webofknowledge.com, Clarivate Analytics, Philadelphia, PA, USA) using the subject search of “plant phenolic induction” and spanning the years of 2008 through 2017 were selected for initial screening. Addition articles were discovered by recovering relevant articles that were referenced by the initially selected articles, as well as using tools to discover articles that used selected articles as a reference. All of these were reduced to 83 by focusing on articles that observed phenolic induction by microorganisms or insects, were original research, and had data suitable for meta-analysis (means and deviations could be obtained or estimated), with the article list provided as Supplementary Data 1. A total of 51 articles were included for meta-analyses on total phenolic levels (Supplementary Table 1), 27 manuscripts for total flavonoid levels (Supplementary Table 2), and 14 for total HCAs levels (Supplementary Table 3). Of these, 17 were shared between total phenolics and flavonoids, 8 were shared between total phenolics and HCAs, 13 shared between flavonoids and HCAs, and 5 shared between all categories.

Roughly one-quarter of the studies had multiple measurements, with either a time course or with different plants interacting with one organism. When different times of measurement were made, a time-point was selected for meta-data that was generally representative of the maximum induction (or a plateau) during the study, as the objective of our meta-analyses was the potential that phenolics would be increased over controls at least for some time period, and not to examine temporal dynamics. With studies with multiple plants evaluated in the publication, each individual plant host was deemed to be a separate study for the meta-analysis. Likewise, separate organisms that induced plant phenolic responses were considered separate trials or studies. In some studies, beneficials and pathogens were used in the same experiment. For these, the beneficial-only (e.g., plant growth-promoting rhizobacteria or mycorrhizae) and pathogen-only controls were used, whereas the combination treatment beneficial-inoculated plants that were later infected by the pathogen) was excluded. Supplementary Tables 1–3 provide details about the data used, including time sampled (if known), host plant, and the organism that may have induced phenolics.

For all studies, means and associated standard errors were used directly if reported, estimated from error bars on graphs, or estimated from reported means separations (as described by Paul et al., 2007). From these, a log-ratio response [L_i_ = ln (μ_Ti_/μ_Ci_); whereas log-ratio response is Li, the mean compound concentration for treated plants is μ_Ti_, and the mean compound concentration for control plants is μ_Ci_], or the log change in phenolic levels from treated vs. non-treated controls, and associated variance [${\text{S}}_{\text{i}}^{2}$L = (V_i_/n_i_)^*^(1/$\mu_{\text{ci}}^{2}$ + 1/$\mu_{\text{Ti}}^{2}$); whereas the standard deviation of the log-ratio response is Si^2^L, V_i_ is the residual variance, and n_i_ is the number of replicates in each group for study] was calculated using the equations from Paul et al. (2007) and Madden and Paul (2011). Percent changes in phenolic levels, along with associated lower and upper confidence intervals, were calculated for mean log-ratio responses using the equation %_change_ = 100^*^(e^Li−1^).

All statistics were performed by SAS version 9.4 (SAS Institute, Cary, NC, USA) using custom macros provided by Madden et al. (2016). Studies with total phenolics, flavonoids, and HCAs were analyzed separately. For each compound type, Forest plots were made of the log-ratio responses per study (sorted by the maximum positive response to maximum negative response), followed by meta-analysis. Forest plots showing calculated log response ratios for each case and separated by insect or microorganism group are shown in Supplementary Figures 1, 2 for total phenolics, Supplementary Figures 3, 4 for total flavonoids, and Supplementary Figures 5, 6 for total HCAs. Meta-analyses consisted of random-effects restricted (residual) maximum likelihood (REML) mixed model analysis of variance (GLIMMIX procedure in SAS) method with the Kenward-Roger adjustment to standard errors and degrees of freedom, with study identity (the article the study cases were within) included in the model as a random factor. REML was chosen to explicitly consider among-study variability in effect size (McNeish and Stapleton, 2016; McNeish, 2017). The Kenward-Roger adjustment further assisted with correcting for the number of studies being small to moderate, and for high variations in sample variances (Kenward and Roger, 1997; McNeish, 2017). Heterogeneity in the meta-analyses was calculated using the *H*^2^ (equivalent to total variability relative to variability under homogeneity) and *I*^2^ (equivalent to the percent of total variability that is due to among-study heterogeneity) statistics, as suggested by Higgins and Thompson (2002) and Higgins et al. (2003), albeit in these cases large calculated heterogeneity in the statistics was interpreted as justification to explicitly use a random-effects model over other approaches (Madden et al., 2016). For these meta-analyses, mean log-ratio responses with 95% confidence intervals >0 indicate a significantly greater phenolic levels in insect infested or microorganism colonized plants compared to controls. Significance was confirmed when a t-statistic had a *p* < 0.05. Categorical moderator variables were included in the procedure to compare and contrast effects (type of biological stressor for all data, biocontrol vs. pathogen for bacteria or fungi, or type of insect), with least-square means used to separate effect estimates from each category. Two such moderators, plant type and biological inducer type, were conducted as independent meta-analyses as these were available for all compound types. More specific moderators, such as insect feeding type or microorganism lifestyle (beneficial or pathogen), had only the relevant study cases included in the analyses. When appropriate, the interaction term of kingdom of the micro-organism (bacteria or fungi) by micro-organism lifestyle (beneficial or pathogen) was assessed in the model. To assess publication bias and sensitivity, funnel plots for the meta-analyses were generated and tested via the “trim and fill” method as described by Duval and Tweedie (2000a,b), using the “rightmost run” estimator R_0_. The final “trimmed and filled” dataset had the meta-analyses re-run and if conclusions (significant effect or not) matched the original analyses, it was assumed that publication bias was acceptable (Duval and Tweedie, 2000b). Additionally, Cook's D was calculated for all log ratio metadata to identify data that highly influenced results, with identified log ratios removed when necessary when determined as outliers (Cook, 1979; Bollen and Jackman, 1990). When conclusions after outlier removal were similar to those with no outlier removal, it was concluded that outlier could remain within the analyses. To address time-lag bias, plots were made of cumulative grand mean effect sizes (as log-ratio responses) proceeding from the first year included in these meta-analyses (2008) through the final year (2017) for total phenolics, flavonoids, and HCAs, similar to as performed by Moreira et al. (2018). Finally, bubble plots with regressions to assess journal impact factor bias by plotting calculated log-ratio responses against journal impact factors, as suggested by Koricheva and Gurevitch (2014) and Nakagawa et al. (2017).

## Results

### Overall Phenolic Compound Induction in Response to Insects or Microorganisms

For total phenolic levels, the mean log-ratio responses across all biological attackers was 0.3741 and significantly >0 with a confidence interval (CI) of 0.2423–0.5058 (*t* = 5.71; *p* < 0.0001; *N* = 111). *H*^2^ was calculated to be 132.669 for the REML (Higgins and Thompson, 2002), whereas *I*^2^ was calculated to be 99.2462 (Higgins et al., 2003), confirming the need to explicitly consider the case study as a random factor in the analysis. Likewise, for total flavonoids log-ratio responses were significantly >0 with an estimate of 0.4531 (CI of 0.2010–0.7052) (*t* = 3.69; *p* = 0.0010; *N* = 82; *H*^2^ = 11.8130; *I*^2^ = 91.5347). This also was the case for total HCAs as the mean estimate log-ratio response was 0.8137 (CI of 0.3744–1.2530) (*t* = 4.13; *p* = 0.0020; *N* = 51; *H*^2^ = 12.1354; *I*^2^ = 91.7597).

**Figure 3:**
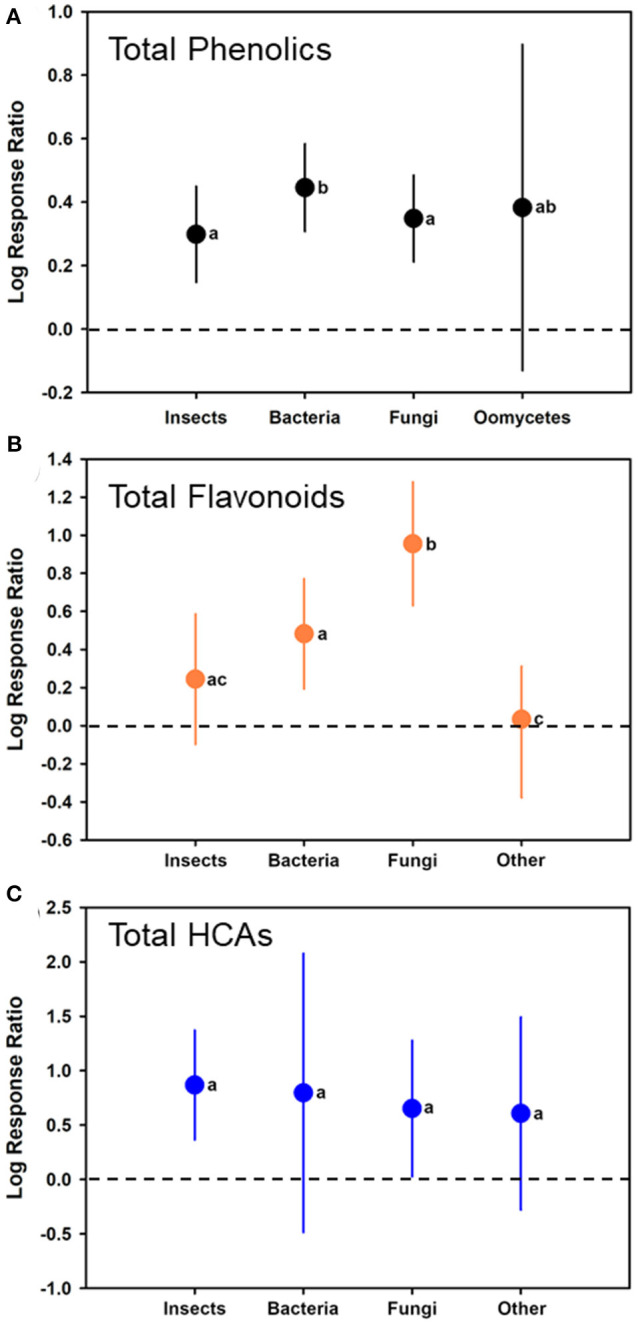
Mean (± 95% confidence interval) log response ratios representing changes in **(A)** total phenolic levels, **(B)** total flavonoid levels, or **(C)** total HCA levels from those in control plants with those feed upon insects, colonized by bacteria, colonized by fungi, or colonized by oomycetes/a combination of other pathogens). In cases where the confidence intervals did not intersect with 0, it was considered that the inducer caused significant changes. Different letters represent differences among the inducer groups by least significant difference (LSD) tests.

As a moderator, the type of attack/colonizer had a significant effect (*p* < 0.05) on the model (*F*_3, 107_ = 6.490; *p* = 0.0004). Comparing log ratio responses among the different potential inducers, bacteria significantly (estimate of 0.4456, with CI of 0.3075–0.5836) had greater induction than insects (estimate of 0.2985 with CI of 0.1475–0.4494) and fungi (estimate of 0.3483 with CI of 0.2120–0.4845), but no differences when compared to oomycetes (estimate of 0.3828 with CI of −0.1307 to 0.8963) (Table 1, Figure 3A). No other differences were observed (separated by comparing least squares means).

**Table 1:** Summary of the meta-analyses to answer four research questions about plant reliance on phenolic compound induction following insect feeding or microorganism colonization.

**Research question**	**Phenolic class**	**Inducer/Plant type**	***K***	***N***	***t***	***P***	**% change**	**CI lower**	**CI upper**
Do phenolic levels increase in response to insects or microorganisms?	Total phenolics	All	111	51	5.71	<0.0001	45.37	27.42	65.83
		Insects	25	12	3.94	0.0002	34.78	15.89	56.75
		Bacteria	38	25	6.48	<0.0001	56.14	36.00	79.25
		Fungi	43	21	5.14	<0.0001	41.67	23.61	62.34
		Oomycetes	5	3	1.50	0.1400	46.64	−12.25	145.05
	Flavonoids	All	82	31	3.69	0.0010	57.32	22.26	102.43
		Insects	27	9	1.43	0.1581	27.66	−9.34	79.75
		Bacteria	24	16	3.39	0.0018	61.98	21.30	116.30
		Fungi	22	9	5.90	<0.0001	159.97	87.87	259.74
		Other Microbes	9	6	−0.19	0.8480	−3.27	−31.50	36.59
	Hydroxycinnamic acids	All	51	18	4.13	0.0020	125.62	45.41	250.08
		Insects	27	8	1.74	0.0883	138.14	43.92	294.01
		Bacteria	11	9	1.31	0.2074	121.49	−38.50	697.73
		Fungi	9	7	2.17	0.0415	91.94	2.80	258.37
		Other Microbes	4	2	1.43	0.1688	83.42	32.13	344.51
Do phenolic level changes differ depending on whether plants were colonized with beneficial microorganisms or pathogens?	Total phenolics	Beneficial Bacteria	24	16	4.84	<0.001	73.78	21.84	147.86
		Pathogenic Bacteria	14	7	3.24	0.0039	78.21	39.21	128.12
		Beneficial Fungi	15	8	4.96	<0.0001	63.44	33.54	100.07
		Pathogenic Fungi	28	18	1.71	0.1006	16.91	−3.65	41.79
Do phenolic level changes differ depending on how insects feed on plants?	Total Phenolics	Chewing Insects	11	5	2.72	0.0272	60.46	7.17	140.25
		Piercing–Sucking Insects	9	5	−0.09	0.9297	−1.70	−36.91	53.16
		Wood-Boring Insects	5	2	−1.01	0.3385	−24.88	−60.47	42.78
Do phenolic level changes differ whether infested/inoculated plants are annuals or perennials?	Total Phenolics	Annuals	68	35	5.81	<0.0001	72.86	31.80	126.73
		Perennials	43	16	1.88	0.0662	38.85	5.15	83.33
	Flavonoids	Annuals	51	20	4.12	0.0003	78.53	35.50	135.23
		Perennials	31	11	2.40	0.0220	42.28	7.22	88.78
	Hydroxycinnamic Acids	Annuals	38	12	3.54	0.0060	126.42	34.61	280.80
		Perennials	13	6	1.65	0.1371	122.55	−27.13	579.64

*Meta-analyses number of case studies used in each category (K), number of corresponding articles (N), t-statistics, and p-values (P) are provided, as well as the mean percent change in phenolic levels in induced plants compared to controls [with 95% confidence intervals (CI)]*.

For total flavonoids and HCAs, induction in the plants was similar to that when total phenolics were observed (Figures 3B,C, respectively). For total flavonoid levels, bacterial (*t* = 3.39; *p* = 0.0018; *N* = 24; log-ratio estimate = 0.4823 [CI of 0.1931–0.7715]) and fungal (*t* = 5.90; *p* < 0.0001; *N* = 22; log-ratio estimate = 0.9554 [CI of 0.6306–1.2802]) colonization had log ratios significantly >0, whereas insect feeding (*t* = 1.43; *p* = 0.1581; *N* = 27; log-ratio estimates is 0.2442 [CI of −0.09807 to 0.5864]) and other microbes with a log-ratio estimate of −0.03325 (CI of −0.3783 to 0.3118) (*t* = −0.19; *p* = 0.8480; *N* = 9) colonization did not. Fungal infection had a greater flavonoid response than all other inducers, and bacterial infection had great induction than other microbes (generally nematodes) (*F*_3, 78_ = 12.12; *p* < 0.0001) (Figure 3B). For total HCAs, log ratios were estimated as 0.8677 (CI of 0.3641–1.3712) for insect feeding (*t* = 3.78; *p* = 0.0029; *N* = 27), 0.7952 (CI of −0.4862 to 2.0766) for colonization by bacteria (*t* = 1.31; *p* = 0.2074; *N* = 11), 0.6520 (CI of 0.02758–1.2764) for colonization by fungi (*t* = 2.17; *p* = 0.0415; *N* = 9), and 0.6066 (CI of −0.2786 to 1.4918) for colonization by other microbes (*t* = 1.43; *p* = 0.1688; *N* = 4). Log response ratios did not differ among the groups for total HCAs (*F*_3, 47_ = 0.43; *p* = 0.7327) (Figure 3C).

### Differences in Phenolic Induction Between Beneficial and Pathogen Colonization

There were only enough cases to compare changes of total phenolics caused by beneficials compared to pathogens. The beneficials did not have significantly greater induction than pathogens (*F*_1, 76_ = 1.24; *p* = 0.2718). There was no significant effect of microorganism kingdom (bacteria or fungal) (*F*_1, 76_ = 3.77; *p* = 0.0594). The lifestyle by kingdom interaction also was not significant (*F*_3, 76_ = 0.13; *p* = 0.7217). The overall REML model returned estimates of a log response ratio of 0.3748 (CI from 0.2171–0.5326) (*t* = 4.820; *p* < 0.0001; *H*^2^ = 161.548; *I*^2^ = 99.3810). Overall, beneficials significantly increased phenolic levels (estimate of 0.3248 with a CI range of 0.1501–0.4996, significantly >0 with *t* = 3.77 and *p* = 0.0006). For pathogens this also was the case (0.4371 with a CI range of 0.2370–0.6373, with *t* = 4.43 and *p* < 0.0001).

**Figure 4:**
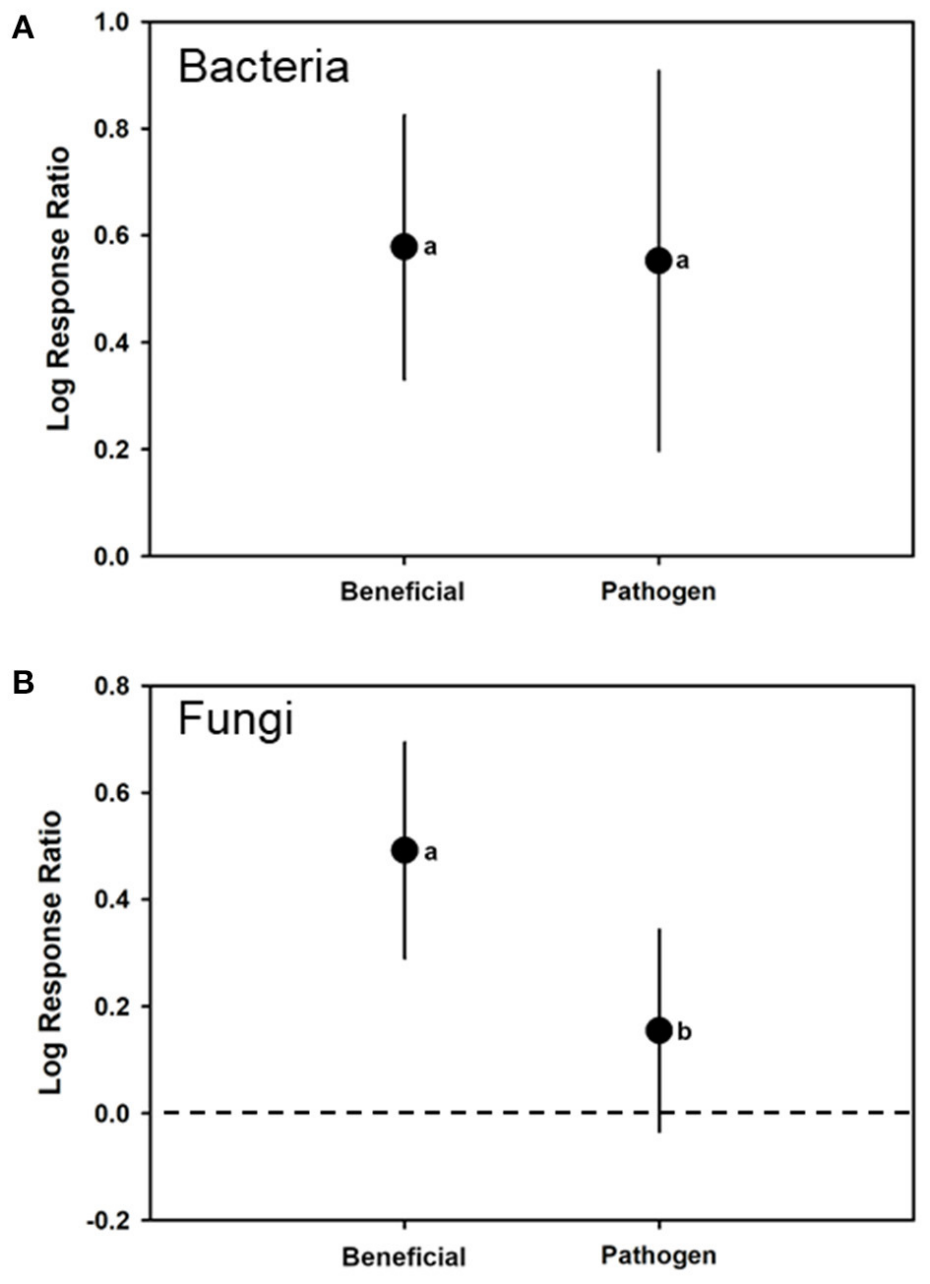
Mean (± 95% confidence interval) log response ratios representing changes in total phenolic levels in control plants with those colonized by **(A)** bacterial or **(B)** fungal biological control agents or pathogens. In cases where the confidence intervals did not intersect with 0, it was considered that the inducer caused significant cases. Different letters represent differences among the inducer groups by least significant difference (LSD) tests.

For bacteria, the overall REML analyses had a mean log ratio estimate of 0.5694 (CI of 0.3716–0.7672) (*H*^2^ = 22.3928; *I*^2^ = 95.5343). As moderators, both bacterial beneficials [log-ratio response estimate of 0.5788 (CI of 0.3308–0.8247) (*t* = 4.84; *p* < 0.0001; *N* = 24) and bacterial pathogens with a log-ratio response estimate of 0.5526 (CI of 0.1975–0.9077) (*t* = 3.24; *p* < 0.0039; *N* = 14) had log response ratios >0, suggesting that colonization by either would increase plant total phenolic levels (Figure 4A). However, there was no significant difference in log response ratios by type of bacteria (*F*_1, 36_ = 0.010; *p* = 0.9050) (Table 1).

For fungi, the overall REML analyses had a mean log ratio estimate of 0.2539 (CI of 0.06281–0.4450) (*H*^2^ = 249.436; *I*^2^ = 99.5991). Fungal beneficials had a log-ratio response estimate of 0.4920 (0.2902–0.6938) (*t* = 4.97; *p* < 0.0001; *N* = 15) and fungal pathogens with a log-ratio response estimate 0.1547 (−0.03413 to 0.34350) (*t* = 1.70; *p* = 0.1036; *N* = 28). In both cases, log-ratio responses were significantly >0 suggesting colonization by either increased phenolic levels (Figure 4B). There was a significant difference for log response ratio due to fungal lifestyle (*F*_1, 41_ = 32.220; *p* < 0.0001) with beneficials have a greater log response ratio than the pathogens (Table 1).

### Differences in Phenolic Induction Due to Different Types of Insect Feeding

**Figure 5:**
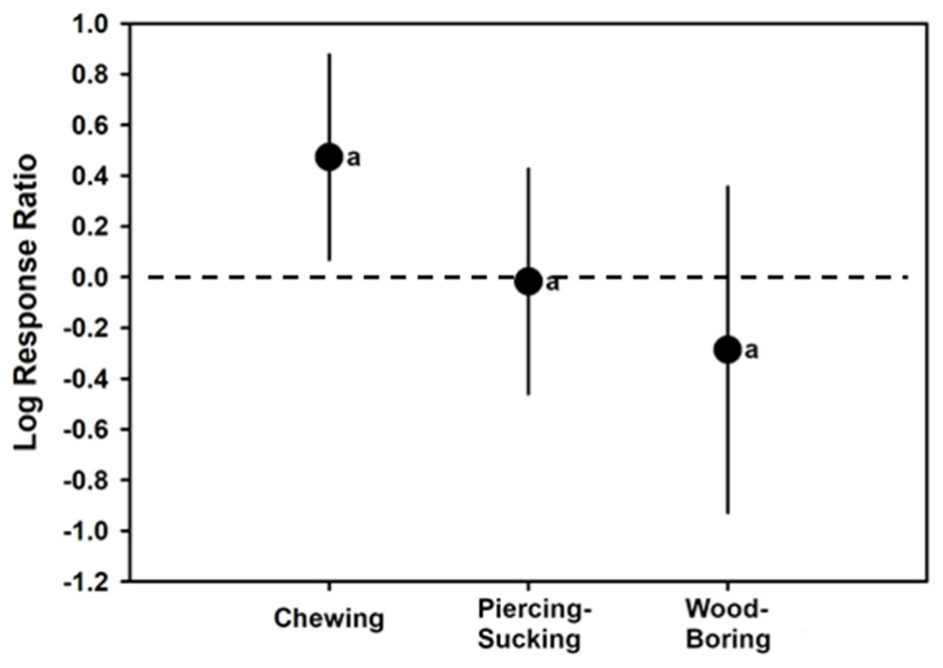
Mean (± 95% confidence interval) log response ratios representing changes in total phenolic levels in control plants with those colonized by chewing, piercing-sucking, or wood-boring insects. In cases where the confidence intervals did not intersect with 0, it was considered that the inducer caused significant cases. Different letters represent differences among the inducer groups by least significant difference (LSD) tests.

There were not enough cases to assess insect feeding type altering plant total flavonoid or HCA levels. However, different insect feeding styles Affecting induction of total phenolic levels in plants was able to be assessed as a moderator. The overall REML model for insect feeding on total phenolics had a mean log-ratio response of 0.1632 (CI of −0.1526 to 0.4789) (*t* = 1.17; *p* = 0.2726; *H*^2^ = 54.5962; *I*^2^ = 98.1684). Chewing insects had total phenolic log-ratio response estimates significantly >0, with an estimate of 0.4729 (CI of 0.06924–0.8765) (*t* = 2.72; *p* = 0.0272; *N* = 11). However, insects that fed by piercing-sucking (*t* = −0.09; *p* = 0.9297; *N* = 9) with a log-ratio response estimate of −0.01716 (CI of −0.4606 to 0.4263), or wood-boring insects (*t* = −1.01; *p* = 0.3385; *N* = 5) with a log-ratio response estimate of −0.2861 (CI of −0.9282 to 0.3561) did not have log response ratios different than 0. In other words, based on these meta-analyses chewing insects increased plant total phenolic levels, whereas piercing-sucking or wood-boring insects did not. However, log-ratio responses were not significantly (*p* < 0.05) greater for chewing insects than insects that those that fed differently (*F*_2, 21_ = 3.31; *p* = 0.0907) (Figure 5, Table 1).

### Differences in Phenolic Induction in Annual vs. Perennial Plants

**Figure 6:**
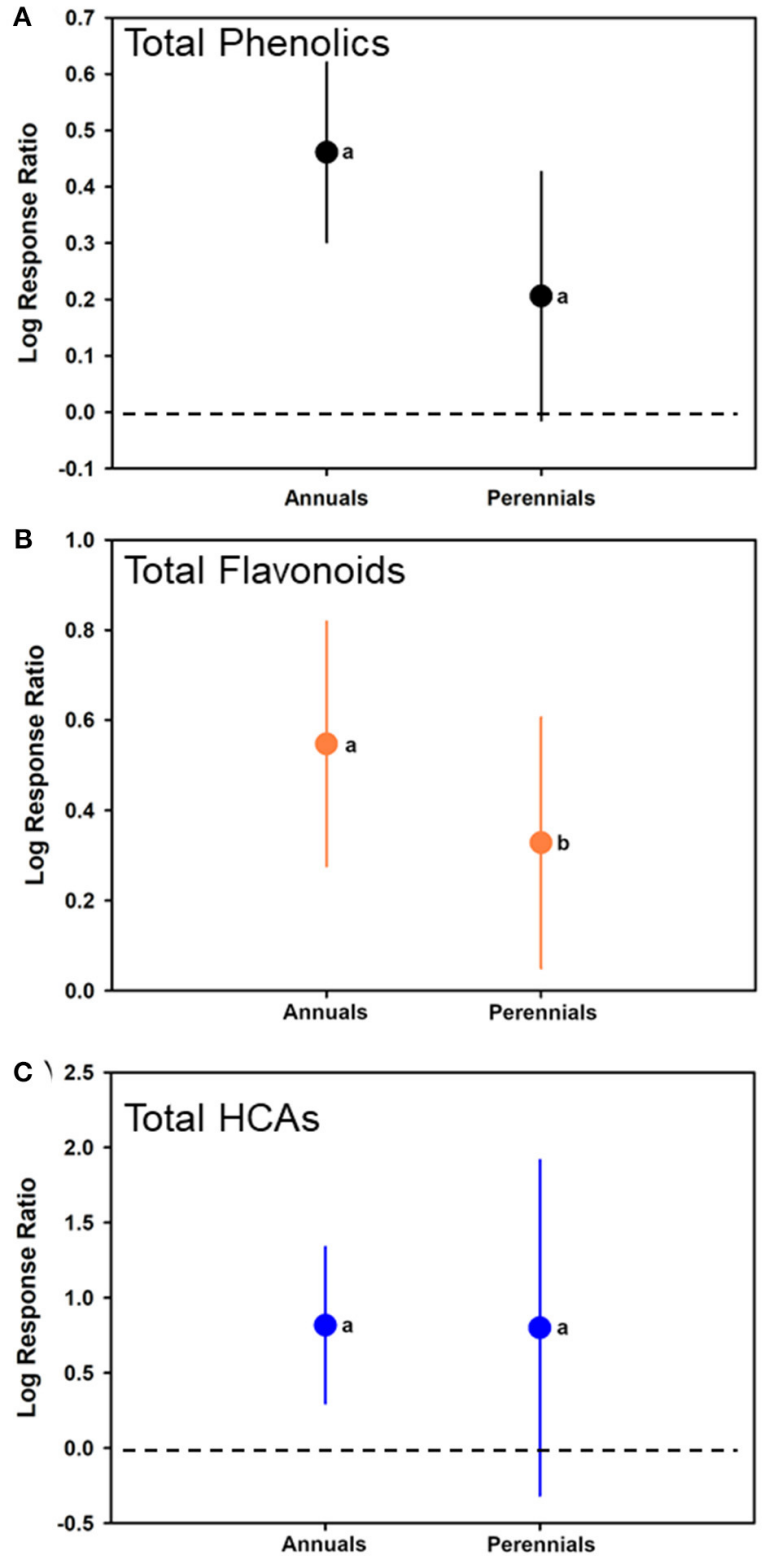
Mean (± 95% confidence interval) log response ratios representing changes in **(A)** total phenolic levels, **(B)** total flavonoid levels, or **(C)** total HCA levels from those in control plants with those induced, with plants separated based on whether they were annuals or perennials. Different letters represent differences among the inducer groups by least significant difference (LSD) tests.

An additional moderator, plant growth habit (annual or perennial) was included in the model. For total phenolics, no significant differences were determined between annual and perennials in terms of phenolic responses (*F*_1, 109_ = 3.560; *p* = 0.0654). Log-ratio responses were significantly >0 for annuals with an estimate of 0.4611 (CI of 0.3016–0.6205) (*t* = 0.4611, *p* < 0.001; *N* = 68), but not significantly different than 0 for perennials with an estimate of 0.2060 (CI of −0.01437 to 0.4264) (*t* = 0.2060; *p* = 0.0662; *N* = 43) (Figure 6A).

This also was the case for total flavonoids with an estimate of 0.5473 (CI of 0.2761–0.8186) for annuals (*t* = 4.12, *p* = 0.0003; *N* = 51), and 0.3282 (CI of 0.05026–0.6061) for perennials (*t* = 2.40; *p* = 0.0220; *N* = 31) (Figure 6B). Annuals had greater log-ratio response estimates than perennials (*F*_1, 80_ = 5.91; *p* = 0.0178). For total HCAs, annuals had a log-ratio response estimate of 0.8172 (CI of 0.2972–1.3371) (*t* = 3.54, *p* = 0.0060; *N* = 38), and perennials had a log-ratio response estimate of 0.8000 (CI of −0.3165 to 1.9164) (*t* = 1.65; *p* = 0.1371; *N* = 13) (Figure 6C, Table 1). For HCAs, there was no difference in the log-ratio responses of annuals or perennials (*F*_1, 49_ = 0.00; *p* = 0.9752). Forest plots showing calculated log response ratios for each case and separated by annual or perennial are shown in Supplementary Figure 4 for total phenolics, Supplementary Figure 5 for total flavonoids, and Supplementary Figure 6 for total HCAs.

### Publication Bias and Sensitivity Analyses

Visual assessments of funnel plots were conducted to determine possible publication biases. For total phenolics, visual assessment suggested that six studies potentially skewed results to have a greater induction than otherwise (Supplementary Figure 7A). To account for this potential publication bias, the “trim and fill” approach was utilized according to Duval and Tweedie (2000b), whereby additional, reciprocal log-ratios of these six were added to the dataset and the meta-analyses was re-run (the modified funnel plot is given in Supplementary Figure 7B). The results on the total phenolic meta-analyses after trim and fill reached similar conclusions (i.e., the inducers positively increased phenolic levels), with the estimated mean of 0.2679 (CI of 0.1624–0.3735) (*H*^2^ = 77.8301; *I*^2^ = 98.7152). Due to the similarity with the unaltered dataset, we concluded that publication bias was minimum, which was the primary interpretation to be reached from the trim and fill method according to (Duval and Tweedie, 2000b). A plot of the restricted likelihood distance from these analyses revealed three potential cases with influences on L greater than all other cases via Cook's D for the total phenolics meta-analyses. All three cases were deleted to assess impact of these potential outliers, and the meta-analyses were re-run to observe the maximum impact that inclusion would have on results with an estimated mean of 0.3799 and CI ranging from 0.2327 to 0.5271 (*H*^2^ = 166.845; *I*^2^ = 99.4006), which would not affect conclusions.

Funnel plots and Cook's distance analyses also were examined for flavonoid and HCA analyses, as well as for the moderator analyses (Supplementary Figures 8, 9), and in all cases publication bias was not present or did not affect conclusions. Likewise, no outliers were identified.

Observations of temporal trends of cumulated grand mean effect sizes demonstrated that effects stabilized for total phenolics around 2012, for flavonoids around 2011, and for HCAs around 2013 (Supplementary Figure 10). Correlations of journal impact factor for each case study with effect sizes did not suggest significant trends for total phenolics, flavonoids, or HCAs (Supplementary Figure 11).

## Discussion

Across all treatments, total phenolics levels, flavonoids, and HCAs were increased in plants attacked or colonized by any biological inducer. Attack by oomycetes and non-bacterial/fungal microbes did not result in significant phenolic effects as detectable by these meta-analyses. However, poor representation of studies likely influenced these conclusions (nine or less cases for inductions of total phenolics, flavonoids, and hydroxycinnamic acid derivatives). Clearly, future studies are warranted to examine phenolic induction by oomycetes, nematodes, parasitic plants, and viruses, albeit a handful of studies suggests induction is expected (Wallis and Sudarshana, 2016; Wallis, 2020).

Despite consistent overall trends in total phenolics and flavonoids, the use of moderators revealed a more complicated picture, including potential differences in insect feeding style and fungal lifestyle on the induction of phenolics. When investigating the general relationship of a microorganism with its host plant (beneficial or pathogenic), it was anticipated that phenolic induction would be higher in plants colonized with beneficial microorganisms vs. those colonized by pathogens, when compared to their respective, untouched controls. The classification of type of bacteria infecting plants did not differ, as both beneficial and pathogenic bacteria had meta-analyses suggesting a significant increase in plant phenolic levels following colonization. In contrast, beneficial fungi caused a significant induction of phenolics, while pathogenic fungi did not. In another meta-analyses study, similar results were found with endophytic/biotrophic vs. necrotrophic fungal challenges on host plants leading to the latter increasing insect performance on the host plant (Fernandez-Conradi et al., 2018). In this study, both the beneficial bacteria and fungi as well as arguably bacterial pathogens could be classified as “biotrophs” and consistently induced phenolic production, but fungal pathogens, for which many were necrotrophic, did not. Therefore, phenolic inductions could have played a role in conclusions reached by Fernandez-Conradi et al. (2018) regarding pathogen effects on insect performance, albeit more direct observations are still warranted. Indeed, necrotrophic fungal pathogens likely cause an increase in unanalyzed sugars and other nutrients, via cell-wall degradation and other enzymatic activity, in the plant that might benefit insect performance (Cardoza et al., 2003; Johnson et al., 2003). Furthermore, in most studies only an introduced bacteria or fungi was considered influencing the plant host even though it was extremely likely that many microbes comprised a community within or on the plant prior to inoculation of the study organism. The roles of the community as a whole on observations of phenolic inductions warrant study, something that is undergoing increased interest (Partida-Martinez and Heil, 2011).

Our third hypothesis was that differences in how insects feed on plants would impact host phenolic responses. Albeit no significant differences were discovered in estimated phenolic log-ratio responses among insect feeding type. It was observed that chewing insect attacks caused significantly greater phenolic levels than non-treated plants, but this was not the case for piercing-sucking or wood-boring insect attacks. Induced resistance to chewing insects is normally associated with jasmonic acid-associated (JA) resistance, whereas piercing-sucking insects would be associated with salicylic acid-associated (SA) resistance (Spoel et al., 2007; Ali and Agrawal, 2012; Thaler et al., 2012; Al-Naemi and Hatcher, 2013). Phenolic production was often considered to be associated with SA-resistance responses, but these results, along with other studies (Vogt, 2010; Yang et al., 2011; Yan et al., 2018; Islam et al., 2019) suggest it may be induced by JA. This finding was somewhat consistent with Fernandez-Conradi et al. (2018), who did not observe insect feed guild differences in observed responses to fungal infections in plants. Likewise, Moreira et al. (2018), who examined JA- and SA-signaling in plants induced by chewing insects, sucking insects, necrotrophic pathogens, and biotrophic pathogens on subsequent attacker inducing JA or SA pathways. They concluded that reciprocal antagonism between pathways was not universal. The findings from these meta-analyses provide partial support that this may be the case, as phenolics, which could potentially impact host resistance to insects and pathogens, did not appear to be significantly different in induction due to insect feeding guild or microorganism lifestyle. At the very least, total phenolics were unaffected by inducers, if not positively increased, suggesting no suppression occurring via one pathway compared to another. More studies investigating the specific phenolics increased or decreased during different insect feeding may elucidate some differences in phenolics that are observed in the phytohormone pathways.

It should be noted that for piercing-sucking insects, the use of salivary sheaths may actively inhibit or reduce the triggering of host defense responses (Will et al., 2013). This is likely an evolutionary strategy by these insects to allow for long-term feeding necessary to acquire nutrients. Because phenolic compounds are most often increased following attack during the hypersensitive response that subsequently causes cell death, insects that need living tissues and homeostatic plant physiology for successful feeding would be better served to inhibit host defense responses. For wood-boring insects, it is possible that the combination of their own feeding with the co-colonization of fungal pathogens that they harbor overwhelms the host defense system, resulting in no measurable changes in levels of phenolics (Erbilgin et al., 2006).

Our final hypothesis was that perennials would rely greater on phenolic induction than annual plants, due to the need for inducible protection to maintain health over a much longer time. This was observed to not be the case, as annual and perennials had increases in phenolics in response to insect or microorganism colonization statically equally. In fact, phenolic log-ratio responses trended higher for annuals in all three categories (total phenolics, flavonoids, and HCAs). It would be easy to argue that insect feeding guild and type of microbe perhaps influenced induction far more than plant type. For instance, all wood-boring insects would attack perennials and not annuals, and this feeding guild did not increase phenolic levels. Likewise, perhaps other compounds or phenolic types (i.e., condensed tannins in cell walls) would be relied on in perennial plants but do not exist in annuals. Regardless, these meta-analyses were not able to assess whether this was the case and additional studies are warranted to elucidate trends for annuals vs. perennials, preferentially investigating phenolic levels in more detail and in similar tissue types (i.e., leaves). In addition, assessments of ontogeny on phenolic production are warranted, as changes in levels during plant development would impact phenolic levels complicating observations on compound induction.

Among final considerations, this effort to analyze a host biochemical response to infection or infestation revealed some gaps in literature and considerations need to be made in future research. Studies examining host physiological responses to many major pathogen groups are sorely lacking, by directly assessing phytocompound levels and not only transcripts of pathway genes, in particular those observing how host plants react to infections by nematodes (Kaplan et al., 2008a,b; Singh et al., 2012; Alves et al., 2016), oomycetes (Sahoo et al., 2009; Korgan et al., 2011), parasitic plants (Runyon et al., 2008; Tjiurutue et al., 2016), and viruses/viroids (Harish et al., 2009; Baker et al., 2010; Wallis and Sudarshana, 2016). The ability to fully assess whether these compounds have consistent responses is considered to remain unknown.

In addition, to enable meta-analyses on host responses future experiments should strive to collect and share data in a consistent manner. The develop of appropriate conventions to use in future studies are necessary to observe consistent responses across a variety of factors, whether different plants, microbes, or insects. Indeed, the quite substantial variation in these meta-analyses due to random factors of study might be attributed to variations in study design. Certain questions still remain after these meta-analyses as well. Specifically, studies about phenolic induction in the future need to include more time-courses, as too few studies were present to have this as a moderator to assess the dynamics of responses. Mouttet et al. (2013) observed differential host resistance affects following fungal infection, and it would be argued that much more work is necessary to observe such trends via meta-analyses.

Likewise, additional biotic factors would be important to include in future studies examining phenolic induction in response to insects or microorganisms. Being able to account for environmental conditions, phenology of the plant, and abiotic stress could go a long way toward accounting for study variability that was concluded in these meta-analyses. In addition, these meta-analyses considered distal (away from induction) phenolic levels only- it would be important to have another meta-analysis from local interactions for comparison.

In conclusion, these meta-analyses confirmed plants, in general, respond to beneficial microbe/pathogen/insect feeding by increasing phenolic compound levels. Yet, in specific cases, such as piercing-sucking or wood-boring insects, no consistent differences in phenolics was observed. It also was likely that certain organisms have evolved to avoid or break down phenolics when feeding to ensure they can require their necessary nutrition to thrive. Regardless, observations of inductions to certain organisms provides support that phenolics compounds are one potential molecular mechanism that plants utilize to protect from attack, albeit studies to directly support this are warranted.

## Data Availability Statement

The datasets are available at the Ag Data Commons of the National Agricultural Library, USDA-ARS (https://data.nal.usda.gov/). The DOI of the datasets is doi: 10.15482/USDA.ADC/1520556.

## Author Contributions

CW conceived the study, compiled the metadata, and conducted the analyses. CW and EG reviewed the results, prepared the manuscript, and performed the necessary editing. All authors contributed to the article and approved the submitted version.

## Conflict of Interest

The authors declare that the research was conducted in the absence of any commercial or financial relationships that could be construed as a potential conflict of interest.
