# Supplementary material for: Phenolic Compound Induction in Plant-Microbe and Plant-Insect Interactions: A Meta-Analysis
Source: Front Plant Sci. 2020 Dec 15;11:580753. doi: 10.3389/fpls.2020.580753 (PMC7769804; doi:10.3389/fpls.2020.580753)
Supplement: Supplementary file 1 [file Data_Sheet_1.DOCX]

Supplementary Material

# Supplementary Data 1. References of the manuscripts used in the meta-analyses.

Aires, A., Dias, C. S. P., Carvalho, R., Oliveira, M. H., Monteiro, A. A., Simoes, M. V., Rosa, E. A. S., Bennett, R. N., and Saavedra, M. J. (2011). Correlations between disease severity, glucosinolate profiles and total phenolics and Xanthomonas campestris pv. campestris inoculation of different Brassicaceae. Sci. Hort. 129, 503-510.

Aksoy, H. M., Kaya, Y., Ozturk, M., Secgin, Z., Onder, H., and Okumus, A. (2017). Pseudomonas putida - Induced response in phenolic profile of tomato seedlings (Solanum lycopersicum L.) infected by Clavibacter michiganensis subsp. michiganensis. Biol. Cont. 105, 6-12.

Alves, G. C. S., Ferri, P. H., Seraphin, J. C., Fortes, G. A. C., Rocha, M. R., and Santos, S. C. (2016). Principal Response Curves analysis of polyphenol variation in resistant and susceptible cotton after infection by a root-knot nematode (RKN). Physiol. Mol. Plant Pathol. 96, 19-28.

Anand, T., Bhaskaran, R., Raguchander, T., Samiyappan, R., Prakasam, V., and Gopalakrishnan, C. (2009). Defence responses of chilli fruits to Colletotrichum capsici and Alternaria alternata. Biol. Plantarum 53, 553-559.

Baker, C. J., Owens, R. A., Whitaker, B. D., Mock, N. M., Roberts, D. P., Deahl, K. L., and Aver'yanov, A. A. (2010). Effect of viroid infection on the dynamics of phenolic metabolites in the apoplast of tomato leaves. Physiol. Mol. Plant Pathol. 74, 214-220.

Buensanteai, N., Yuen, G. Y., and Prathuangwong, S. (2009). Priming, signaling, and protein production associated with induced resistance by Bacillus amyloliquefaciens KPS46. World J. Microbiol. Biotech. 25, 1275-1286.

Cappellari, L. D., Santoro, M. V., Nievas, F., Giordano, W., and Banchio, E. (2013). Increase of secondary metabolite content in marigold by inoculation with plant growth-promoting rhizobacteria. App. Soil Ecol. 70, 16-22.

Carrillo-Gavilan, A., Moreira, X., Zas, R., Vila, M., and Sampedro, L. (2012). Early resistance of alien and native pines against two native generalist insect herbivores: no support for the natural enemy hypothesis. Func. Ecol. 26, 283-293.

Chakraborty, U., Chakraborty, B. N., Basnet, M., and Chakraborty, A. P. (2009). Evaluation of Ochrobactrum anthropi TRS-2 and its talc based formulation for enhancement of growth of tea plants and management of brown root rot disease. J. App. Microbiol. 107, 625-634.

Chakraborty, U., Chakraborty, B. N., and Chakraborty, A. P. (2010). Influence of Serratia marcescens TRS-1 on growth promotion and induction of resistance in Camellia sinensis against Fomes lamaoensis. J. Plant Inter. 5, 261-272.

Chen, Y. G., Whitehill, J. G. A., Bonello, P., and Poland, T. M. (2011a). Differential response in foliar chemistry of three ash species to emerald ash borer adult feeding. J. Chem. Ecol. 37, 29-39.

Chen, Y. G., Whitehill, J. G. A., Bonello, P., and Poland, T. M. (2011b). Feeding by emerald ash borer larvae induces systemic changes in black ash foliar chemistry. Phytochem. 72, 1990-1998.

Cirak, C., Radusiene, J., Aksoy, H. M., Mackinaite, R., Stanius, Z., Camas, N., and Odabas, M. S. (2014). Differential phenolic accumulation in two Hypericum species in response to inoculation with Diploceras hypericinum and Pseudomonas putida. Plant Prot. Sci. 50, 119-128.

Czerniewicz, P., Sytykiewicz, H., Durak, R., Borowiak-Sobkowiak, B., and Chrzanowski, G. (2017). Role of phenolic compounds during antioxidative responses of winter triticale to aphid and beetle attack. Plant Physiol. Biochem. 118, 529-540.

Erbilgin, N., Krokene, P., Christiansen, E., Zeneli, G., and Gershenzon, J. (2006). Exogenous application of methyl jasmonate elicits defenses in Norway spruce (Picea abies) and reduces host colonization by the bark beetle Ips typographus. Oecologia 148, 426-436.

Figueiro, A. D., Reese, N., Hernandez, J. L. G., Pacheco, M. T., Martinelli, J. A., Federizzi, L. C., and Delatorre, C. A. (2015). Reactive oxygen species are not increased in resistant oat genotypes challenged by crown rust isolates. J. Phytopathol. 163, 795-806.

Ge, Y., Guest, D. I., and Bi, Y. (2014). Differences in the induction of defence responses in resistant and susceptible muskmelon plants Infected with Colletotrichum lagenarium. J. Phytopathol. 162, 48-54.

Glazebrook, J. (2005). Contrasting mechanisms of defense against biotrophic and necrotrophic pathogens. Ann. Rev. Phytopathol. 43, 205-227.

Golan, K., Sempruch, C., Gorska-Drabik, E., Czerniewicz, P., Lagowska, B., Kot, I., Kmiec, K., Magierowicz, K., and Leszczynski, B. (2017). Accumulation of amino acids and phenolic compounds in biochemical plant responses to feeding of two different herbivorous arthropod pests. Arthropod-Plant Inter. 11, 675-682.

Guerrero-Molina, M. F., Lovaisa, N. C., Salazar, S. M., Martinez-Zamora, M. G., Diaz-Ricci, J. C., and Pedraza, R. O. (2015). Physiological, structural and molecular traits activated in strawberry plants after inoculation with the plant growth-promoting bacterium Azospirillum brasilense REC3. Plant Biol. 17, 766-773.

Gupta, R., and Pandey, R. (2015). Microbial interference ameliorates essential oil yield and diminishes root-knot infestation in sweet basil under field conditions. Biocontrol Sci. Tech. 25, 1165-1179.

Gutbrodt, B., Mody, K., Wittwer, R., and Dorn, S. (2011). Within-plant distribution of induced resistance in apple seedlings: rapid acropetal and delayed basipetal responses. Planta 233, 1199-1207.

Harish, S., Kavino, M., Kumar, N., Balasubramanian, P., and Samiyappan, R. (2009). Induction of defense-related proteins by mixtures of plant growth promoting endophytic bacteria against Banana bunchy top virus. Biol. Cont. 51, 16-25.

Hassan, M. A. E., and Abo-Elyousr, K. A. M. (2013). Activation of tomato plant defence responses against bacterial wilt caused by Ralstonia solanacearum using DL-3-aminobutyric acid (BABA). Eur. J. Plant Pathol. 136, 145-157.

Jain, A., Singh, S., Sarma, B. K., and Singh, H. B. (2012). Microbial consortium-mediated reprogramming of defence network in pea to enhance tolerance against Sclerotinia sclerotiorum. J. Appl. Microbiol. 112, 537-550.

Jain, A., Singh, A., Singh, S., and Singh, H. B. (2015). Phenols enhancement effect of microbial consortium in pea plants restrains Sclerotinia sclerotiorum. Biol. Cont. 89, 23-32.

Kaplan, I., Halitschke, R., Kessler, A., Sardanelli, S., and Denno, R. F. (2008a). Effects of plant vascular architecture on aboveground-belowground-induced responses to foliar and root herbivores on Nicotiana tabacum. J. Chem. Ecol. 34, 1349-1359.

Kaplan, I., Halitschke, R., Kessler, A., Sardanelli, S., and Denno, R. F. (2008b). Constitutive and induced defenses to herbivory in above- and belowground plant tissues. Ecology 89, 392-406.

Kaur, R., Gupta, A. K., and Taggar, G. K. (2014). Role of catalase, H2O2 and phenolics in resistance of pigeonpea towards Helicoverpa armigera (Hubner). Acta Physiol. Plantarum 36, 1513-1527.

Kaur, H., Salh, P. K., and Singh, B. (2017). Role of defense enzymes and phenolics in resistance of wheat crop (Triticum aestivum L.) towards aphid complex. J. Plant Inter. 12, 304-311.

Khatun, S., and Chatterjee, N. C. (2011). Glomus fasciculatum in defense responses to fusarial wilt of Coleus forskohlii. Acta Agricul. Scandinavica Sect. B- Soil Plant Sci. 61, 136-142.

Kiprovski, B., Malencic, D., Popovic, M., Stojsin, V., Budakov, D., Curcic, Z., and Danojevic, D. (2014). Correlation between lipid peroxidation and phenolics content in leaves and roots of sugar beet infected with Rhizoctonia solani. Phytoparasitica 42, 199-203.

Konappa, N. M., Maria, M., Uzma, F., Krishnamurthy, S., Nayaka, S. C., and Niranjana, S. R. (2016). Lactic acid bacteria mediated induction of defense enzymes to enhance the resistance in tomato against Ralstonia solanacearum causing bacterial wilt. Sci. Hort. 207, 183-192.

Korgan, S., Wolski, E. A., Cicore, P., Suarez, P., Capezio, S., Huarte, M. A., and Andreu, A. B. (2011). Solanum tarijense reaction to Phytophthora infestans and the role of plant defence molecules. Plant Breed. 130, 231-236.

Latha, P., Anand, T., Rappathi, N., Prakasam, V., and Samiyappan, R. (2009). Antimicrobial activity of plant extracts and induction of systemic resistance in tomato plants by mixtures of PGPR strains and Zimmu leaf extract against Alternaria solani. Biol. Cont. 50, 85-93.

Malolepsza, U., Nawrocka, J., and Szczech, M. (2017). Trichoderma virens 106 inoculation stimulates defence enzyme activities and enhances phenolic levels in tomato plants leading to lowered Rhizoctonia solani infection. Biocont. Sci. Tech. 27, 180-199.

Mamani, A., Filippone, M. P., Grellet, C., Welin, B., Castagnaro, A. P., and Ricci, J. C. D. (2012). Pathogen-induced accumulation of an ellagitannin elicits plant defense response. Mol.-Plant Microbe Inter. 25, 1430-1439.

Martins, S. J., de Medeiros, F. H. V., de Souza, R. M., de Resende, M. L. V., and Ribeiro, P. M. (2013). Biological control of bacterial wilt of common bean by plant growth-promoting rhizobacteria, Biol. Cont. 66, 65-71.

Mauch-Mani, B., and Metraux, J. (1998). Salicylic acid and systemic acquired resistance to pathogen attack. Ann. Bot. 82, 535-540.

Metraux, J., Nawrath, C., and Genoud, T. (2002). Systemic acquired resistance. Euphytica 124, 237-243.

Mollavali, M., Bolandnazar, S. A., Schwarz, D., Rohn, S., Riehle, P., and Nahandi, F. Z. (2016). Flavonol glucoside and antioxidant enzyme biosynthesis affected by mycorrhizal fungi in various cultivars of onion (Allium cepa L.). J. Agri. Food Chem. 64, 71-77.

Moreira, X., Abdala-Roberts, L., Hernandez-Cumplido, J., Cuny, M. A. C., Glauser, G., and Benrey, B. (2015). Specificity of induced defenses, growth, and reproduction in lima bean (Phaseolus lunatus) in response to multispecies herbivory. Amer. J. Bot.102, 1300-1308.

Muthukumar, A., and Venkatesh, A. (2014). Biological inductions of systemic resistance to collar rot of peppermint caused by Sclerotium rolfsii. Acta Physiol. Plantarum 36, 1421-1431.

Ortega-Garcia, J. G., Montes-Belmont, R., Rodriguez-Monroy, M., Ramirez-Trujillo, J. A., Suarez-Rodriguez, R., and Sepulveda-Jimenez, G. (2015). Effect of Trichoderma asperellum applications and mineral fertilization on growth promotion and the content of phenolic compounds and flavonoids in onions. Sci. Hort. 195, 8-16.

Pascual-Alvarado, E., Cuevas-Reyes, P., Quesada, M., and Oyama, K. (2008). Interactions between galling insects and leaf-feeding insects: the role of plant phenolic compounds and their possible interference with herbivores. J. Trop. Ecol. 24, 329-336.

Raffa, K. F., Mason, C. J., Bonello, P., Cook, S., Erbilgin, N., Keefover-Ring, K., Klutsch, J. G., Villari, C., and Townsend, P. A. (2017). Defence syndromes in lodgepole - whitebark pine ecosystems relate to degree of historical exposure to mountain pine beetles. Plant Cell Environ. 40, 1791-1806.

Ramos, O. F., Smith, C. M., Fritz, A. K., and Madl, R. L. (2017). Bird-cherry oat aphid (Rhopalosiphum padi) feeding stress induces enhanced levels of phenolics in mature wheat grains. Crop Sci. 57, 2073-2079.

Ray, S., Mondal, S., Chowdhury, S., and Kundu, S. (2015). Differential responses of resistant and susceptible tomato varieties to inoculation with Alternaria solani. Physiol. Mol. Plant Pathol. 90, 78-88.

Reis, F. S., Ferreira, I. C. F. R., Barros, L., Santos-Buelga, C., and Martins, A. (2011). Mycorrhizal induction of phenolic compounds and antioxidant properties of fungi and seedlings during the early steps of symbiosis. Chemoecol. 21, 151-159.

Rudikovskaya, E. G., Akimova, G. P., Fedorova, G. A., Sokolova, M. G., Dudareva, L. V., and Rudikovskii, A. V. (2010). Dynamics of accumulation of phenolic compounds in pea roots during interaction with symbiotic bacteria Rhizobium leguminosarum. Rus. J. Plant Physiol. 57, 253-259.

Runyon, J.B., Mescher, M. C., and DeMoraes, C. M. (2008). Parasitism by Cuscuta pentagona attenuates host plant defenses against insect herbivores. Plant Physiol. 146, 987–995.

Sahoo, M. R., Kole, P. C., Dasgupta, M., and Mukherjee, A. (2009). Changes in phenolics, polyphenol oxidase and its isoenzyme patterns in relation to resistance in taro against Phytophthora colocasiae. J. Phytopathol. 157, 145-153.

Salla, T. D., da Silva, T. R., Astarita, L. V., and Santarem, E. R. (2014). Streptomyces rhizobacteria modulate the secondary metabolism of Eucalyptus plants. Plant Physiol. Biochem. 85, 14-20.

Salla, T. D., Astarita, L. V., and Santarem, E. R. (2016). Defense responses in plants of Eucalyptus elicited by Streptomyces and challenged with Botrytis cinerea. Planta 243, 1055-1070.

Sambangi, P., and Pani, P. U. (2013). Induction of phenolic acids and metals in Arachis hypogaea L. plants due to feeding of three lepidopteran pests. Arthropod-Plant Inter. 7, 517-525.

Sari, E., Etebarian, H. R., and Aminian, H. (2008). Effects of Pseudomonas fluorescens CHA0 on the resistance of wheat seedling roots to the take-all fungus Gaeumannomyces graminis var. tritici. Plant Prot. Sci. 11, 298-306.

Shahbazi, H., Aminian, H., Sahebani, N., and Halterman, D. A. (2010). Biochemical evaluation of resistance responses of potato to different isolates of Alternaria solani. Phytopathol. 100, 454-459.

Shafique, S., Ahmad, A., Shafique, S., Anjum, T., Akram, W., and Bashir, Z. (2014). Determination of molecular and biochemical changes in cotton plants mediated by mealybug. NJAS-Wageningen J. Life Sci. 70-71, 39-45.

Shivashankar, S., Sumathi, M., Krishnakumar, N. K., and Rao, V. K. (2015). Role of phenolic acids and enzymes of phenylpropanoid pathway in resistance of chayote fruit (Sechium edule) against infestation by melon fly, Bactrocera cucurbitae. Ann. Appl. Biol. 166, 420-433.

Singh, A., Jain, A., Sarma, B. K., Upadhyay, R. S., and Singh, H. B. (2014). Beneficial compatible microbes enhance antioxidants in chickpea edible parts through synergistic interactions. LWT- Food Sci. Tech. 56, 390-397.

Singh, S. P., and Gaur, R. (2017). Endophytic Streptomyces spp. underscore induction of defense regulatory genes and confers resistance against Sclerotium rolfsii in chickpea. Biol. Cont. 104, 44-56.

Singh, S. P., Gupta, R., Gaur, R., and Srivastava, A. K. (2016). Streptomyces spp. alleviate Rhizoctonia solani-mediated oxidative stress in Solanum lycopersicon. Ann. Appl. Biol.168, 232-242.

Singh, U. B., Sahu, A., Sahu, N., Singh, R. K., Renu, Prabha, R., Singh, D. R., Sarma, B. K., and Manna, M. C. (2012). Co-inoculation of Dactylaria brochopaga and Monacrosporium eudermatum affects disease dynamics and biochemical responses in tomato (Lycopersicon esculentum Mill.) to enhance bio-protection against Meloidogyne incognita. Crop Prot. 35, 102-109.

Sorahinobar, M., Niknam, V., Ebrahimzadeh, H., Soltanloo, H., Behmanesh, M., and Enferadi, S. T. (2016). Central role of salicylic acid in resistance of wheat against Fusarium graminearum. J. Plant Growth Reg. 35, 477-491.

Thaler, J. S., Fidantsef, A. L., Duffey, S. S., and Bostock, R. M. (1999). Trade-offs in plant defense against pathogens and herbivores: a field demonstration of chemical elicitors of induced resistance. J. Chem. Ecol. 25, 1597–1609.

Tjiurutue, M. C., Sandler, H. A., Kersch-Becker, M. F., Theis, N., and Adler, L. A. (2016). Cranberry resistance to dodder parasitism: induced chemical defenses and behavior of a parasitic plant. J. Chem. Ecol. 42, 95-106.

Vallad, G. E., and Goodman, R. M. (2004). Systemic acquired resistance and induced systemic resistance in conventional agriculture. Crop Sci. 44, 1920-1934.

Van Loon, L. C., Bakker, P. A. H. M., and Pieterse, C. M. J. (1998). Systemic resistance induced by rhizosphere bacteria. Ann. Rev. Phytopathol. 36, 453-483.

Vlot, A. C., Dempsey, D. A., and Klessig, D. F. (2009). Salicylic acid, a multifaceted hormone to combat disease. Ann. Rev. Phytopathol. 47, 177-206.

Wallis, C., Eyles, A., Chorbadjian, R., Gardener, B. M., Hansen, R., Cipollini, D., Herms, D. A., and Bonello, P. (2008). Systemic induction of phloem secondary metabolism and its relationship to resistance to a canker pathogen in Austrian pine. New Phytol. 177, 767-778.

Wallis, C. M., and Chen, J. (2012). Grapevine phenolic compounds in xylem sap and tissues are significantly altered during infection by Xylella fastidiosa. Phytopathol. 102, 816-826.

Wallis, C. M., and Sudarshana, M. R. (2016). Effects of Grapevine red blotch-associated virus (GRBaV) infection on foliar metabolism of grapevines. Can. J. Plant Pathol. 38, 358-366.

Wallis, C. M., Chen, J., and Civerolo, E. L. (2012). Zebra chip-diseased potato tubers are characterized by increased levels of host phenolics, amino acids, and defense-related proteins. Physiol. Mol. Plant Pathol. 78, 66-72.

Wallis, C. M., Wallingford, A. K., and Chen, J. (2013). Grapevine rootstock effects on scion sap phenolic levels, resistance to Xylella fastidiosa infection, and progression of Pierce's disease. Front. Plant Sci. 4, 502.

Walters, D., and Heil, M. (2007). Costs and trade-offs associated with induced resistance. Physiol. Mol. Plant Pathol. 71, 3-17.

Wolski, E. A., Henriquez, M. A., Adam, L. R., Badawi, M., Andreu, A. B., El Hadrami, A., and Daayf, F. (2010). Induction of defense genes and secondary metabolites in saskatoons (Amelanchier alnifolia Nutt.) in response to Entomosporium mespili using jasmonic acid and Canada milkvetch extracts. Environ. Exp. Bot. 68, 273-282.

Yashin, N. A., and Ahmed, S. (2016). Induction of defence-related biochemicals by rhizosphere bacteria against black spot disease of rose. Biol. Agri. Hort. 32, 34-46.

Young, B., Wagner, D., Doak, P., and Clausen, T. (2010). Induction of phenolic glycosides by quaking aspen (Populus tremuloides) leaves in relation to extrafloral nectaries and epidermal leaf mining. J. Chem. Ecol. 36, 369-377.

Zeilinger, A. R., Olson, D. M., Maclean, D., Mori, N., Nakata, R., and Andow, D. A. (2015). Behavioural and chemical mechanisms of plant-mediated deterrence and attraction among frugivorous insects. Ecol. Entomol. 40, 532-542.

Zhang, X., Sun, X., Zhao, H., Xue, M., and Wang, D. (2017). Phenolic compounds induced by Bemisia tabaci and Trialeurodes vaporariorum in Nicotiana tabacum L. and their relationship with the salicylic acid signaling pathway. Arthropod-Plant Inter. 11, 659-667.

Supplementary Table 1. Studies and descriptions of cases used for the meta-analysis of changes in plant total phenolic levels following insect attack, pathogen infection, or beneficial colonization.

| Reference | Case | Plant | Plant Type | Organism | Organism Type | Organism Strategy |
| --- | --- | --- | --- | --- | --- | --- |
| Golan et al. 2017 | 1 | *Phalaenopsis* x. hybridum | perennial | *Pseudococcus maritimus* | insect | piercing-sucking |
| Raffa et al. 2017 | 2 | *Pinus concorta* | perennial | *Dendroctonus ponderosae* | insect | wood boring |
| Raffa et al. 2017 | 3 | *Pinus albicaulis* | perennial | *Dendroctonus ponderosae* | insect | wood boring |
| Kaur et al. 2017 | 4 | *Triticum aestivum* | annual | aphids | insect | piercing-sucking |
| Ramos et al. 2017 | 5 | *Triticum aestivum* | annual | *Rhopalosiphum padi* | insect | piercing-sucking |
| Malolepsza et al. 2017 | 6 | *Solanum lycopersicum* | annual | *Rhizoctonia solani* | fungi | pathogen |
| Malolepsza et al. 2017 | 7 | *Solanum lycopersicum* | annual | *Trichoderma virens* | fungi | beneficial |
| Konappa et al. 2016 | 8 | *Solanum lycopersicum* | annual | *Rhizoctonia solani* | fungi | pathogen |
| Konappa et al. 2016 | 9 | *Solanum lycopersicum* | annual | *Lactobacillus paracasei* | bacteria | beneficial |
| Ganapathy et al. 2016 | 10 | *Zingiber officinale* | perennial | *Pythium myriotylum* | oomycete | pathogen |
| Sorahinobar et al. 2016 | 11 | *Triticum aestivum* | annual | *Fusarium graminearum* | fungi | pathogen |
| Sorahinobar et al. 2016 | 12 | *Triticum aestivum* | annual | *Fusarium graminearum* | fungi | pathogen |
| Salla et al. 2016 | 13 | *Eucalyptus grandis* | perennial | *Strepomyces* spp. | bacteria | beneficial |
| Salla et al. 2016 | 14 | *Eucalyptus globulus* | perennial | *Strepomyces* spp. | bacteria | beneficial |
| Salla et al. 2016 | 15 | *Eucalyptus grandis* | perennial | *Botrytis cinerea* | fungi | pathogen |
| Salla et al. 2016 | 16 | *Eucalyptus globulus* | perennial | *Botrytis cinerea* | fungi | pathogen |
| Singh et al. 2016 | 17 | *Solanum lycopersicum* | annual | *Rhizoctonia solani* | fungi | pathogen |
| Mollavali et al. 2016 | 18 | *Allium cepa* | annual | *Funneliformis mosseae* | fungi | beneficial |
| Mollavali et al. 2016 | 19 | *Allium cepa* | annual | *Rhozophagus intraradices* | fungi | beneficial |
| Mollavali et al. 2016 | 20 | *Allium cepa* | annual | *Diversispora versiformis* | fungi | beneficial |
| Yasin et al. 2016 | 21 | *Rosa* spp. | perennial | *Psedumonas fluorescnes* | bacteria | beneficial |
| Yasin et al. 2016 | 22 | *Rosa* spp. | perennial | *Bacillus subtiis* | bacteria | beneficial |
| Yasin et al. 2016 | 23 | *Rosa* spp. | perennial | *Diplocarpon rosae* | fungi | pathogen |
| Ortega-Garcia et al. 2015 | 24 | *Allium cepa* | annual | *Trichoderma asperellum* | fungi | beneficial |
| Ortega-Garcia et al. 2015 | 25 | *Allium cepa* | annual | *Trichoderma harzianum* | fungi | beneficial |
| Figueiro et al. 2015 | 26 | *Avena sativa* | annual | *Puccinia coronata* f. sp. *avenae* | fungi | pathogen |
| Figueiro et al. 2015 | 27 | *Avena sativa* | annual | *Puccinia coronata* f. sp. *avenae* | fungi | pathogen |
| Moreira et al. 2015 | 28 | *Phaseolus lunatus* | annual | *Diabrotica balteata* | insect | chewing |
| Moreira et al. 2015 | 29 | *Phaseolus lunatus* | annual | *Spodoptera eridania* | insect | chewing |
| Guerrero-Molina et al. 2015 | 30 | *Fragaria* ssp. | annual | *Azospirillum brasilense* | bacteria | beneficial |
| Guerrero-Molina et al. 2015 | 31 | *Fragaria* ssp. | annual | *Azospirillum brasilense* | bacteria | beneficial |
| Ray et al. 2015 | 32 | *Solanum lycopersicum* | annual | *Alternaria solani* | fungi | pathogen |
| Ray et al. 2015 | 33 | *Solanum lycopersicum* | annual | *Alternaria solani* | fungi | pathogen |
| Gupta and Pandey 2015 | 34 | *Ocimum basilicum* | annual | *Bacillus subtilis* | bacteria | beneficial |
| Shafique et al. 2014 | 35 | *Gossypium* spp. | annual | *Phenacoccus solenopsis* | insect | piercing-sucking |
| Salla et al. 2014 | 36 | *Eucalyptus* spp. | perennial | *Streptomyces* spp. | bacteria | beneficial |
| Muthukumar and Venkatesh 2014 | 37 | *Mentha* x. *piperita* | perennial | *Pseudomonas fluorescens* | bacteria | beneficial |
| Muthukumar and Venkatesh 2014 | 38 | *Mentha* x. *piperita* | perennial | *Trichoderma harzianum* | fungi | beneficial |
| Muthukumar and Venkatesh 2014 | 39 | *Mentha* x. *piperita* | perennial | *Sclerotium rolfsii* | fungi | pathogen |
| Kaur et al. 2014 | 40 | *Cajanus cajan* | annual | *Helicoverpa armigera* | insect | chewing |
| Singh et al. 2014 | 41 | *Cicer arietinum* | annual | *Mesorhizobium* spp. | bacteria | beneficial |
| Singh et al. 2014 | 42 | *Cicer arietinum* | annual | *Pseudomonas aeruginosa* | bacteria | beneficial |
| Singh et al. 2014 | 43 | *Cicer arietinum* | annual | *Trichoderma harzianum* | fungi | beneficial |
| Kiprovski et al. 2014 | 44 | *Ipomoea batatas* | annual | *Rhizoctonia solani* | fungi | pathogen |
| Kiprovski et al. 2014 | 45 | *Ipomoea batatas* | annual | *Rhizoctonia solani* | fungi | pathogen |
| Ge et al. 2014 | 46 | *Cucumis melo* | annual | *Colletotrichum lagenarium* | fungi | pathogen |
| Ge et al. 2014 | 47 | *Cucumis melo* | annual | *Colletotrichum lagenarium* | fungi | pathogen |
| Sambangi and Rani 2013 | 48 | *Arachis hypogaea* | annual | *Amsacta albistriga* | insect | chewing |
| Sambangi and Rani 2013 | 49 | *Arachis hypogaea* | annual | *Aproaerema modicella* | insect | chewing |
| Sambangi and Rani 2013 | 50 | *Arachis hypogaea* | annual | *Spilosoma obliqua* | insect | chewing |
| Cappellari et al. 2013 | 51 | *Tagetes minuta* | annual | *Azospirillum brasilense* | bacteria | beneficial |
| Cappellari et al. 2013 | 52 | *Tagetes minuta* | annual | *Pseudomonas fluorescens* | bacteria | beneficial |
| Martins et al. 2013 | 53 | *Phaseolus vulgaris* | annual | *Curtobacterium flaccumfaciens* pv. *flaccumfaciens* | bacteria | pathogen |
| Martins et al. 2013 | 54 | *Phaseolus vulgaris* | annual | *Bacillus subtilis* | bacteria | beneficial |
| Hassan and Abo-Elyousr 2013 | 55 | *Solanum lycopersicum* | annual | *Ralstonia solanacearum* | bacteria | pathogen |
| Mamani et al. 2012 | 56 | *Fragaria* ssp. | annual | *Colletotrichum fragariae* | fungi | pathogen |
| Mamani et al. 2012 | 57 | *Fragaria* ssp. | annual | *Colletotrichum acutatum* | fungi | pathogen |
| Wallis and Chen 2012 | 58 | *Vitis vinifera* | perennial | *Xylella fastidiosa* | bacteria | pathogen |
| Wallis and Chen 2012 | 59 | *Vitis vinifera* | perennial | *Xylella fastidiosa* | bacteria | pathogen |
| Wallis et al. 2012 | 61 | *Solanum tuberosum* | annual | *"Ca. Liberibacter solanacearum"* | bacteria | pathogen |
| Jain et al. 2012 | 62 | *Pisum sativa* | annual | *Bacillus subtilis* | bacteria | beneficial |
| Jain et al. 2012 | 63 | *Pisum sativa* | annual | *Pseudomonas aeruginosa* | bacteria | beneficial |
| Jain et al. 2012 | 64 | *Pisum sativa* | annual | *Trichoderma harzianum* | fungi | beneficial |
| Jain et al. 2012 | 65 | *Pisum sativa* | annual | *Sclerotinia sclerotiorum* | fungi | pathogen |
| Carrillo-Gavilan et al. 2012 | 66 | *Pinus pinaster* | perennial | *Hylobius abietis* | insect | chewing |
| Carrillo-Gavilan et al. 2012 | 67 | *Pinus radiata* | perennial | *Hylobius abietis* | insect | chewing |
| Carrillo-Gavilan et al. 2012 | 68 | *Pinus pinaster* | perennial | *Thaumetopoea pityocampa* | insect | chewing |
| Carrillo-Gavilan et al. 2012 | 69 | *Pinus radiata* | perennial | *Thaumetopoea pityocampa* | insect | chewing |
| Reis et al. 2011 | 70 | *Pinus pinaster* | perennial | *Paxillus involutus* | fungi | beneficial |
| Reis et al. 2011 | 71 | *Pinus pinaster* | perennial | *Pisolithus arhizus* | fungi | beneficial |
| Aires et al. 2011 | 72 | *Lepidium sativum* | annual | *Xanthomonas campestris* pv. c*ampestris* | bacteria | pathogen |
| Aires et al. 2011 | 73 | *Eruca sativa* | annual | *Xanthomonas campestris* pv. c*ampestris* | bacteria | pathogen |
| Aires et al. 2011 | 74 | *Brassica oleracea* | annual | *Xanthomonas campestris* pv. c*ampestris* | bacteria | pathogen |
| Aires et al. 2011 | 75 | *Brassica oleracea* | annual | *Xanthomonas campestris* pv. c*ampestris* | bacteria | pathogen |
| Aires et al. 2011 | 76 | *Brassica oleracea* | annual | *Xanthomonas campestris* pv. c*ampestris* | bacteria | pathogen |
| Korgan et al. 2011 | 77 | *Solanum tarijense* | annual | *Phytophora infestans* | oomycete | pathogen |
| Korgan et al. 2011 | 78 | *Solanum tarijense* | annual | *Phytophora infestans* | oomycete | pathogen |
| Khatun and Chatterjee 2011 | 79 | *Coleus forskohlii* | annual | *Glomus fasciculatum* | fungi | beneficial |
| Khatun and Chatterjee 2011 | 80 | *Coleus forskohlii* | annual | *Fusarium oxysporum* | fungi | pathogen |
| Chen et al. 2011a | 81 | *Fraxinus nigra* | perennial | *Agrilus planipennis* | insect | wood boring |
| Chen et al. 2011a | 82 | *Fraxinus pennsylvanica* | perennial | *Agrilus planipennis* | insect | wood boring |
| Chen et al. 2011a | 83 | *Fraxinus americana* | perennial | *Agrilus planipennis* | insect | wood boring |
| Shahbazi et al. 2010 | 84 | *Solanum tuberosum* | annual | *Alternaria solani* | fungi | pathogen |
| Shahbazi et al. 2010 | 85 | *Solanum tuberosum* | annual | *Alternaria solani* | fungi | pathogen |
| Rudikovskaya et al. 2010 | 86 | *Pisum sativa* | annual | *Rhizobium leguminosarum* | bacteria | beneficial |
| Chakraborty et al. 2010 | 87 | *Camellia sinensis* | perennial | *Serriatia marcescens* | bacteria | beneficial |
| Chakraborty et al. 2010 | 88 | *Camellia sinensis* | perennial | *Serriatia marcescens* | bacteria | beneficial |
| Chakraborty et al. 2010 | 89 | *Camellia sinensis* | perennial | *Fomes lamaoensis* | fungi | pathogen |
| Chakraborty et al. 2010 | 90 | *Camellia sinensis* | perennial | *Fomes lamaoensis* | fungi | pathogen |
| Harish et al. 2010 | 91 | *Musa* spp. | perennial | *Bacillus subtilis* | bacteria | beneficial |
| Harish et al. 2010 | 92 | *Musa* spp. | perennial | *Pseudomonas fluorescens* | bacteria | beneficial |
| Anand et al. 2009 | 94 | *Capsicum annum* | annual | *Colletotrichum capsici* | fungi | pathogen |
| Anand et al. 2009 | 95 | *Capsicum annum* | annual | *Alternaria alternata* | fungi | pathogen |
| Latha et al. 2009 | 96 | *Solanum lycopersicum* | annual | *Bacillus subtilis* | bacteria | beneficial |
| Latha et al. 2009 | 97 | *Solanum lycopersicum* | annual | *Pseudomonas fluorescens* | bacteria | beneficial |
| Latha et al. 2009 | 98 | *Solanum lycopersicum* | annual | *Alternaria solani* | fungi | pathogen |
| Chakraborty et al. 2009 | 99 | *Camellia sinensis* | perennial | *Ochrobactrum anthropi* | bacteria | beneficial |
| Chakraborty et al. 2009 | 100 | *Camellia sinensis* | perennial | *Phellinus noxius* | fungi | pathogen |
| Buensanteai et al. 2009 | 101 | *Glycine max* | annual | *Xanthomonas axonopodis* pv. *glycines* | bacteria | pathogen |
| Buensanteai et al. 2009 | 102 | *Glycine max* | annual | *Bacillus amyloliquefaciens* | bacteria | beneficial |
| Sahoo et al. 2009 | 103 | *Colocasia esculenta* | perennial | *Phytophora colocasiae* | oomycete | pathogen |
| Sahoo et al. 2009 | 104 | *Colocasia esculenta* | perennial | *Phytophora colocasiae* | oomycete | pathogen |
| Sari et al. 2008 | 105 | *Triticum aestivum* | annual | *Pseudomonas fluorescens* | bacteria | beneficial |
| Sari et al. 2008 | 106 | *Triticum aestivum* | annual | *Gaeumannomyces graminis* var. *tritici* | fungi | pathogen |
| Pascual-Alvarado et al. 2008 | 107 | *Achatocarpus gracilis* | perennial | galling insects | insect | piercing-sucking |
| Pascual-Alvarado et al. 2008 | 108 | *Cordia alliodora* | perennial | galling insects | insect | piercing-sucking |
| Pascual-Alvarado et al. 2008 | 109 | *Guapira macrocarpa* | perennial | galling insects | insect | piercing-sucking |
| Pascual-Alvarado et al. 2008 | 110 | *Guettarda elliptica* | perennial | galling insects | insect | piercing-sucking |
| Pascual-Alvarado et al. 2008 | 111 | *Ruprechtia fusca* | perennial | galling insects | insect | piercing-sucking |
| Wallis et al. 2008 | 112 | *Pinus nigra* | perennial | *Diplodia pinea* | fungi | pathogen |
| Wallis et al. 2008 | 113 | *Pinus nigra* | perennial | *Neodiprion sertifer* | insect | chewing |

Supplementary Table 2. Studies and descriptions of cases used for the meta-analysis of changes in plant flavonoid levels following insect attack, pathogen infection, or beneficial colonization.

| Reference | Case | Plant | Plant Type | Organism | Organism Type | Organism Strategy |
| --- | --- | --- | --- | --- | --- | --- |
| Zhang et al. 2017 | 1 | *Nicotiana tabacum* | annual | *Bemisia tabaci* | insect | piercing-sucking |
| Zhang et al. 2017 | 2 | *Nicotiana tabacum* | annual | *Trialerurodes vaporariorum* | insect | piercing-sucking |
| Zhang et al. 2017 | 3 | *Nicotiana tabacum* | annual | *Bemisia tabaci* | insect | piercing-sucking |
| Zhang et al. 2017 | 4 | *Nicotiana tabacum* | annual | *Trialerurodes vaporariorum* | insect | piercing-sucking |
| Zhang et al. 2017 | 5 | *Nicotiana tabacum* | annual | *Bemisia tabaci* | insect | piercing-sucking |
| Zhang et al. 2017 | 6 | *Nicotiana tabacum* | annual | *Trialerurodes vaporariorum* | insect | piercing-sucking |
| Zhang et al. 2017 | 7 | *Nicotiana tabacum* | annual | *Bemisia tabaci* | insect | piercing-sucking |
| Zhang et al. 2017 | 8 | *Nicotiana tabacum* | annual | *Trialerurodes vaporariorum* | insect | piercing-sucking |
| Czerniewicz et al. 2017 | 9 | x *Triticosecale* spp. | annual | *Sitobion avenae* | insect | piercing-sucking |
| Czerniewicz et al. 2017 | 10 | x *Triticosecale* spp. | annual | *Oulema melanopus* | insect | chewing |
| Czerniewicz et al. 2017 | 11 | x *Triticosecale* spp. | annual | *Sitobion avenae* | insect | piercing-sucking |
| Czerniewicz et al. 2017 | 12 | x *Triticosecale* spp. | annual | *Oulema melanopus* | insect | chewing |
| Czerniewicz et al. 2017 | 13 | x *Triticosecale* spp. | annual | *Sitobion avenae* | insect | piercing-sucking |
| Czerniewicz et al. 2017 | 14 | x *Triticosecale* spp. | annual | *Oulema melanopus* | insect | chewing |
| Raffa et al. 2017 | 15 | *Pinus contorta* | perennial | *Dendroctonus ponderosae* | insect | wood boring |
| Raffa et al. 2017 | 16 | *Pinus albicaulis* | perennial | *Dendroctonus ponderosae* | insect | wood boring |
| Aksoy et al. 2017 | 17 | *Solanum lycopersicum* | annual | *Pseudomonas putida* | bacteria | beneficial |
| Aksoy et al. 2017 | 18 | *Solanum lycopersicum* | annual | *Clavibacter michiganensis* subsp. *michiganensis* | bacteria | pathogen |
| Malolepsza et al. 2017 | 19 | *Solanum lycopersicum* | annual | *Trichoderma virens* | fungi | beneficial |
| Malolepsza et al. 2017 | 20 | *Solanum lycopersicum* | annual | *Rhizoctonia solani* | fungi | pathogen |
| Malolepsza et al. 2017 | 21 | *Solanum lycopersicum* | annual | *Trichoderma virens* | fungi | beneficial |
| Malolepsza et al. 2017 | 22 | *Solanum lycopersicum* | annual | *Rhizoctonia solani* | fungi | pathogen |
| Singh and Gaur 2017 | 23 | *Cicer arietinum* | annual | *Sclerotium rolsii* | fungi | pathogen |
| Singh and Gaur 2017 | 24 | *Cicer arietinum* | annual | *Streptomyces griseus* | bacteria | beneficial |
| Singh and Gaur 2017 | 25 | *Cicer arietinum* | annual | *Streptomyces olivochromongenes* | bacteria | beneficial |
| Ganapathy et al. 2016 | 26 | *Zingiber officinale* | perennial | *Pythium myriotylum* | oomycete | pathogen |
| Salla et al. 2016 | 27 | *Eucalyptus grandis* | perennial | *Strepomyces* spp. | bacteria | beneficial |
| Salla et al. 2016 | 28 | *Eucalyptus grandis* | perennial | *Botrytis cinerea* | fungi | pathogen |
| Salla et al. 2016 | 29 | *Eucalyptus globulus* | perennial | *Strepomyces* spp. | bacteria | beneficial |
| Salla et al. 2016 | 30 | *Eucalyptus globulus* | perennial | *Botrytis cinerea* | fungi | pathogen |
| Tjiurutue et al. 2016 | 31 | *Vaccinium oxycoccos* | perennial | *Cuscuta* spp. | parasitic plant | pathogen |
| Tjiurutue et al. 2016 | 32 | *Vaccinium oxycoccos* | perennial | *Cuscuta* spp. | parasitic plant | pathogen |
| Wallis and Sudarshana 2016 | 33 | *Vitis vinifera* | perennial | *Grapevine red blotch associated virus* | virus | pathogen |
| Wallis and Sudarshana 2016 | 34 | *Vitis vinifera* | perennial | *Grapevine red blotch associated virus* | virus | pathogen |
| Wallis and Sudarshana 2016 | 35 | *Vitis vinifera* | perennial | *Grapevine red blotch associated virus* | virus | pathogen |
| Ortega-Garcia et al. 2015 | 36 | *Allium cepa* | annual | *Trichoderma asperellum* | fungi | beneficial |
| Ortega-Garcia et al. 2015 | 37 | *Allium cepa* | annual | *Trichoderma harzianum* | fungi | beneficial |
| Zeilinger et al. 2015 | 38 | *Gossypium hirsutum* | annual | *Heliothis virescens* | insect | chewing |
| Zeilinger et al. 2015 | 39 | *Gossypium hirsutum* | annual | *Helicoverpa zea* | insect | chewing |
| Zeilinger et al. 2015 | 40 | *Gossypium hirsutum* | annual | *Heliothis virescens* | insect | chewing |
| Zeilinger et al. 2015 | 41 | *Gossypium hirsutum* | annual | *Helicoverpa zea* | insect | chewing |
| Jain et al . 2015 | 42 | *Pisum sativa* | annual | *Bacillus subtilis* | bacteria | beneficial |
| Jain et al . 2015 | 43 | *Pisum sativa* | annual | *Trichoderma harzianum* | fungi | beneficial |
| Jain et al . 2015 | 44 | *Pisum sativa* | annual | *Pseudomonas aeruginosa* | bacteria | beneficial |
| Jain et al . 2015 | 45 | *Pisum sativa* | annual | *Sclerotinia sclerotiorum* | fungi | pathogen |
| Gupta and Pandey 2015 | 46 | *Ocimum basilicum* | annual | *Bacillus subtilis* | bacteria | beneficial |
| Salla et al. 2014 | 47 | *Eucalyptus* spp. | perennial | *Streptomyces* spp. | bacteria | beneficial |
| Singh et al. 2014 | 48 | *Cicer arietinum* | annual | *Mesorhizobium* spp. | bacteria | beneficial |
| Singh et al. 2014 | 49 | *Cicer arietinum* | annual | *Trichoderma harzianum* | fungi | beneficial |
| Singh et al. 2014 | 50 | *Cicer arietinum* | annual | *Pseudomonas aeruginosa* | bacteria | beneficial |
| Kiprovski et al. 2014 | 51 | *Ipomoea batatas* | annual | *Rhizoctonia solani* | fungi | pathogen |
| Kiprovski et al. 2014 | 52 | *Ipomoea batatas* | annual | *Rhizoctonia solani* | fungi | pathogen |
| Cirak et al. 2014 | 53 | *Hypericum perforatum* | perennial | *Diploceras hypericinum* | fungi | pathogen |
| Cirak et al. 2014 | 54 | *Hypericum perforatum* | perennial | *Pseudonomas putida* | bacteria | beneficial |
| Cirak et al. 2014 | 55 | *Hypericum perforatum* | perennial | *Diploceras hypericinum* | fungi | pathogen |
| Cirak et al. 2014 | 56 | *Hypericum perforatum* | perennial | *Pseudonomas putida* | bacteria | beneficial |
| Ge et al. 2014 | 57 | *Cucumis melo* | annual | *Colletotrichum lagenarium* | fungi | pathogen |
| Ge et al. 2014 | 58 | *Cucumis melo* | annual | *Colletotrichum lagenarium* | fungi | pathogen |
| Wallis et al. 2013 | 59 | *Vitis vinifera* | perennial | *Xylella fastidiosa* | bacteria | pathogen |
| Wallis et al. 2013 | 60 | *Vitis vinifera* | perennial | *Xylella fastidiosa* | bacteria | pathogen |
| Wallis et al. 2013 | 61 | *Vitis vinifera* | perennial | *Xylella fastidiosa* | bacteria | pathogen |
| Wallis et al. 2013 | 62 | *Vitis vinifera* | perennial | *Xylella fastidiosa* | bacteria | pathogen |
| Wallis et al. 2013 | 63 | *Vitis vinifera* | perennial | *Xylella fastidiosa* | bacteria | pathogen |
| Wallis et al. 2013 | 64 | *Vitis vinifera* | perennial | *Xylella fastidiosa* | bacteria | pathogen |
| Wallis and Chen 2012 | 65 | *Vitis vinifera* | perennial | *Xylella fastidiosa* | bacteria | pathogen |
| Wallis and Chen 2012 | 66 | *Vitis vinifera* | perennial | *Xylella fastidiosa* | bacteria | pathogen |
| Wallis et al. 2012 | 67 | *Solanum tuberosum* | annual | *"Ca. Liberibacter solanacearum"* | bacteria | pathogen |
| Gutbrodt et al. 2011 | 68 | *Malus pumila* | perennial | *Spodoptera littoralis* | insect | chewing |
| Gutbrodt et al. 2011 | 69 | *Malus pumila* | perennial | *Spodoptera littoralis* | insect | chewing |
| Wolski et al. 2010 | 70 | *Amelanchier alnifolia* | perennial | *Entomosporium mespili* | fungi | pathogen |
| Wolski et al. 2010 | 71 | *Amelanchier alnifolia* | perennial | *Entomosporium mespili* | fungi | pathogen |
| Wolski et al. 2010 | 72 | *Amelanchier alnifolia* | perennial | *Entomosporium mespili* | fungi | pathogen |
| Wolski et al. 2010 | 73 | *Amelanchier alnifolia* | perennial | *Entomosporium mespili* | fungi | pathogen |
| Rudikovskaya et al. 2010 | 74 | *Pisum sativa* | annual | *Rhizobium leguminosarum* | bacteria | beneficial |
| Kaplan et al. 2008a | 75 | *Nicotiana tabacum* | annual | *Spodoptera exigua* | insect | chewing |
| Kaplan et al. 2008a | 76 | *Nicotiana tabacum* | annual | *Meloidogyne incognita* | nematode | pathogen |
| Kaplan et al. 2008b | 77 | *Nicotiana tabacum* | annual | *Manduca sexta* | insect | chewing |
| Kaplan et al. 2008b | 78 | *Nicotiana tabacum* | annual | *Manduca sexta* | insect | chewing |
| Kaplan et al. 2008b | 79 | *Nicotiana tabacum* | annual | *Trichoplusia ni* | insect | chewing |
| Kaplan et al. 2008b | 80 | *Nicotiana tabacum* | annual | *Trichoplusia ni* | insect | chewing |
| Kaplan et al. 2008b | 81 | *Nicotiana tabacum* | annual | *Meloidogyne incognita* | nematode | pathogen |
| Kaplan et al. 2008b | 82 | *Nicotiana tabacum* | annual | *Meloidogyne incognita* | nematode | pathogen |

Supplementary Table 3. Studies and descriptions of cases used for the meta-analysis of changes in plant phenolic acid levels following insect attack, pathogen infection, or beneficial colonization.

| Reference | Case | Plant | Plant Type | Organism | Organism Type | Organism Strategy |
| --- | --- | --- | --- | --- | --- | --- |
| Zhang et al. 2017 | 1 | *Nicotiana tabacum* | annual | *Bemisia tabaci* | insect | piercing-sucking |
| Zhang et al. 2017 | 2 | *Nicotiana tabacum* | annual | *Trialerurodes vaporariorum* | insect | piercing-sucking |
| Czerniewicz et al. 2017 | 3 | x *Triticosecale* spp. | annual | *Sitobion avenae* | insect | piercing-sucking |
| Czerniewicz et al. 2017 | 4 | x *Triticosecale* spp. | annual | *Oulema melanopus* | insect | chewing |
| Czerniewicz et al. 2017 | 5 | x *Triticosecale* spp. | annual | *Sitobion avenae* | insect | piercing-sucking |
| Czerniewicz et al. 2017 | 6 | x *Triticosecale* spp. | annual | *Oulema melanopus* | insect | chewing |
| Raffa et al. 2017 | 7 | *Pinus contorta* | perennial | *Dendroctonus ponderosae* | insect | wood boring |
| Raffa et al. 2017 | 8 | *Pinus albicaulis* | perennial | *Dendroctonus ponderosae* | insect | wood boring |
| Raffa et al. 2017 | 9 | *Pinus contorta* | perennial | *Dendroctonus ponderosae* | insect | wood boring |
| Raffa et al. 2017 | 10 | *Pinus albicaulis* | perennial | *Dendroctonus ponderosae* | insect | wood boring |
| Raffa et al. 2017 | 11 | *Pinus contorta* | perennial | *Dendroctonus ponderosae* | insect | wood boring |
| Raffa et al. 2017 | 12 | *Pinus albicaulis* | perennial | *Dendroctonus ponderosae* | insect | wood boring |
| Aksoy et al. 2017 | 13 | *Solanum lycopersicum* | annual | *Pseudomonas putida* | bacteria | beneficial |
| Aksoy et al. 2017 | 14 | *Solanum lycopersicum* | annual | *Clavibacter michiganensis* subsp. *michiganensis* | bacteria | pathogen |
| Aksoy et al. 2017 | 15 | *Solanum lycopersicum* | annual | *Pseudomonas putida* | bacteria | beneficial |
| Aksoy et al. 2017 | 16 | *Solanum lycopersicum* | annual | *Clavibacter michiganensis* subsp. *michiganensis* | bacteria | pathogen |
| Tjiurutue et al. 2016 | 17 | *Vaccinium oxycoccos* | perennial | *Cuscuta* spp. | parasitic plant | pathogen |
| Zeilinger et al. 2015 | 18 | *Gossypium hirsutum* | annual | *Heliothis virescens* | insect | chewing |
| Zeilinger et al. 2015 | 19 | *Gossypium hirsutum* | annual | *Helicoverpa zea* | insect | chewing |
| Zeilinger et al. 2015 | 20 | *Gossypium hirsutum* | annual | *Heliothis virescens* | insect | chewing |
| Zeilinger et al. 2015 | 21 | *Gossypium hirsutum* | annual | *Helicoverpa zea* | insect | chewing |
| Jain et al. 2015 | 22 | *Pisum sativa* | annual | *Bacillus subtilis* | bacteria | beneficial |
| Jain et al. 2015 | 23 | *Pisum sativa* | annual | *Trichoderma harzianum* | fungi | beneficial |
| Jain et al. 2015 | 24 | *Pisum sativa* | annual | *Pseudomonas aeruginosa* | bacteria | beneficial |
| Jain et al. 2015 | 25 | *Pisum sativa* | annual | *Sclerotinia sclerotiorum* | fungi | pathogen |
| Jain et al. 2015 | 26 | *Pisum sativa* | annual | *Bacillus subtilis* | bacteria | beneficial |
| Jain et al. 2015 | 27 | *Pisum sativa* | annual | *Trichoderma harzianum* | fungi | beneficial |
| Jain et al. 2015 | 28 | *Pisum sativa* | annual | *Pseudomonas aeruginosa* | bacteria | beneficial |
| Jain et al. 2015 | 29 | *Pisum sativa* | annual | *Sclerotinia sclerotiorum* | fungi | pathogen |
| Jain et al. 2015 | 30 | *Pisum sativa* | annual | *Bacillus subtilis* | bacteria | beneficial |
| Jain et al. 2015 | 31 | *Pisum sativa* | annual | *Trichoderma harzianum* | fungi | beneficial |
| Jain et al. 2015 | 32 | *Pisum sativa* | annual | *Pseudomonas aeruginosa* | bacteria | beneficial |
| Jain et al. 2015 | 33 | *Pisum sativa* | annual | *Sclerotinia sclerotiorum* | fungi | pathogen |
| Shivashankar et al. 2015 | 34 | *Sechium edule* | annual | *Bactrocera cucurbitae* | insect | chewing |
| Shivashankar et al. 2015 | 35 | *Momordica charantia* | annual | *Bactrocera cucurbitae* | insect | chewing |
| Cirak et al. 2014 | 36 | *Hypericum perforatum* | perennial | *Diploceras hypericinum* | fungi | pathogen |
| Cirak et al. 2014 | 37 | *Hypericum perforatum* | perennial | *Pseudonomas putida* | bacteria | beneficial |
| Chen et al. 2011b | 38 | *Fraxinus nigra* | perennial | *Agrilus planipennis* | insect | wood boring |
| Wolski et al. 2010 | 39 | *Amelanchier alnifolia* | perennial | *Entomosporium mespili* | fungi | pathogen |
| Wolski et al. 2010 | 40 | *Amelanchier alnifolia* | perennial | *Entomosporium mespili* | fungi | pathogen |
| Young et al. 2010 | 41 | *Populus tremuloides* | perennial | *Phyllocnistis populiella* | insect | chewing |
| Kaplan et al. 2008a | 42 | *Nicotiana tabacum* | annual | *Spodoptera exigua* | insect | chewing |
| Kaplan et al. 2008a | 43 | *Nicotiana tabacum* | annual | *Spodoptera exigua* | insect | chewing |
| Kaplan et al. 2008a | 44 | *Nicotiana tabacum* | annual | *Spodoptera exigua* | insect | chewing |
| Kaplan et al. 2008a | 45 | *Nicotiana tabacum* | annual | *Meloidogyne incognita* | nematode | pathogen |
| Kaplan et al. 2008b | 46 | *Nicotiana tabacum* | annual | *Manduca sexta* | insect | chewing |
| Kaplan et al. 2008b | 47 | *Nicotiana tabacum* | annual | *Manduca sexta* | insect | chewing |
| Kaplan et al. 2008b | 48 | *Nicotiana tabacum* | annual | *Trichoplusia ni* | insect | chewing |
| Kaplan et al. 2008b | 49 | *Nicotiana tabacum* | annual | *Trichoplusia ni* | insect | chewing |
| Kaplan et al. 2008b | 50 | *Nicotiana tabacum* | annual | *Meloidogyne incognita* | nematode | pathogen |
| Kaplan et al. 2008b | 51 | *Nicotiana tabacum* | annual | *Meloidogyne incognita* | nematode | pathogen |

**
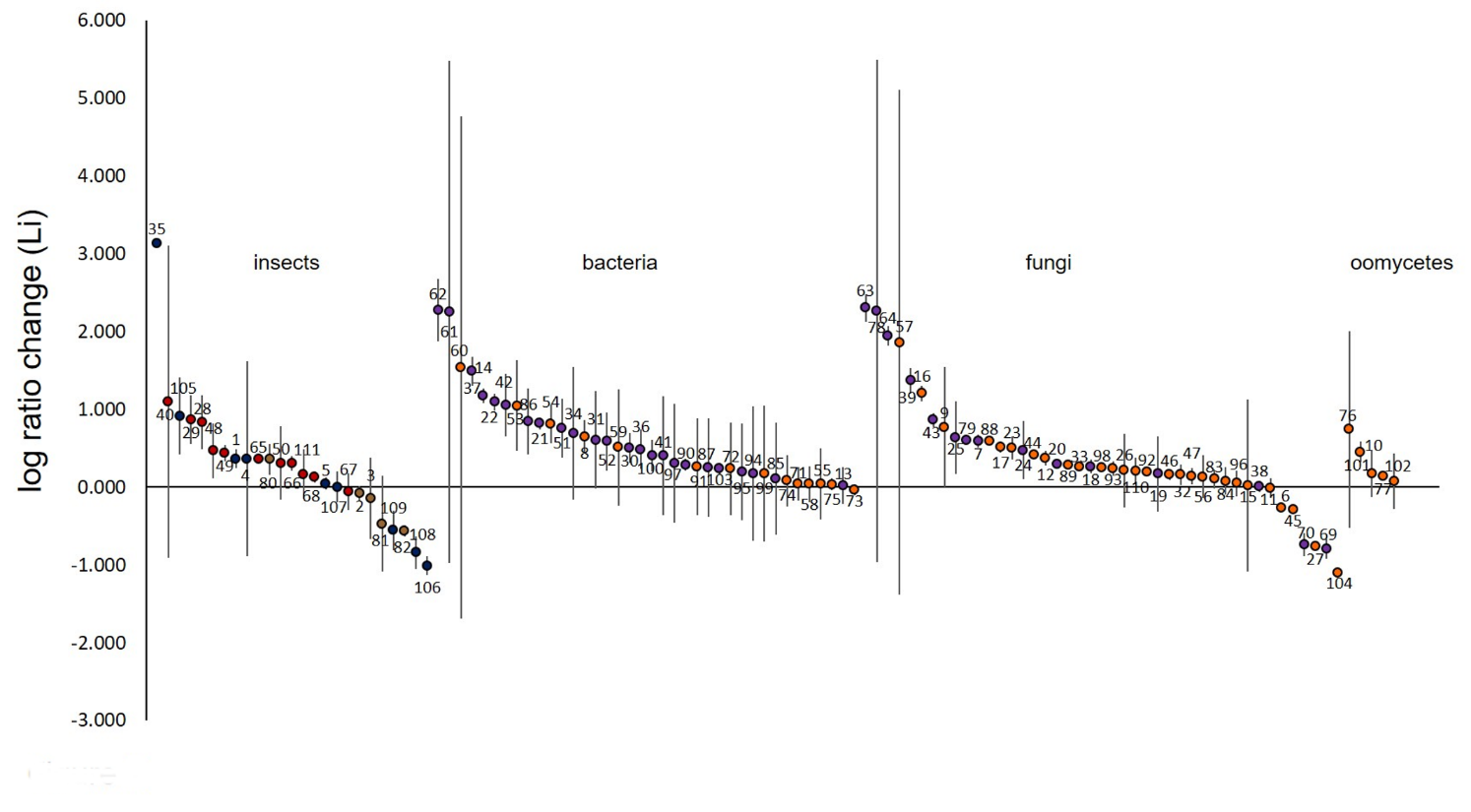
**

**Supplementary Figure 1.** Forest plot for log-ratio (L) representing change for total phenolics for each case separated by insect or pathogen type. For insects, red represents chewing insects, blue represents piercing-sucking insects, and brown represents wood-boring insects. For microbes, purple represents biological control agents and orange represents pathogens. Numbers above each point correspond to case number listed in Supplementary Table 1. Bars represent 95% confidence intervals.

**
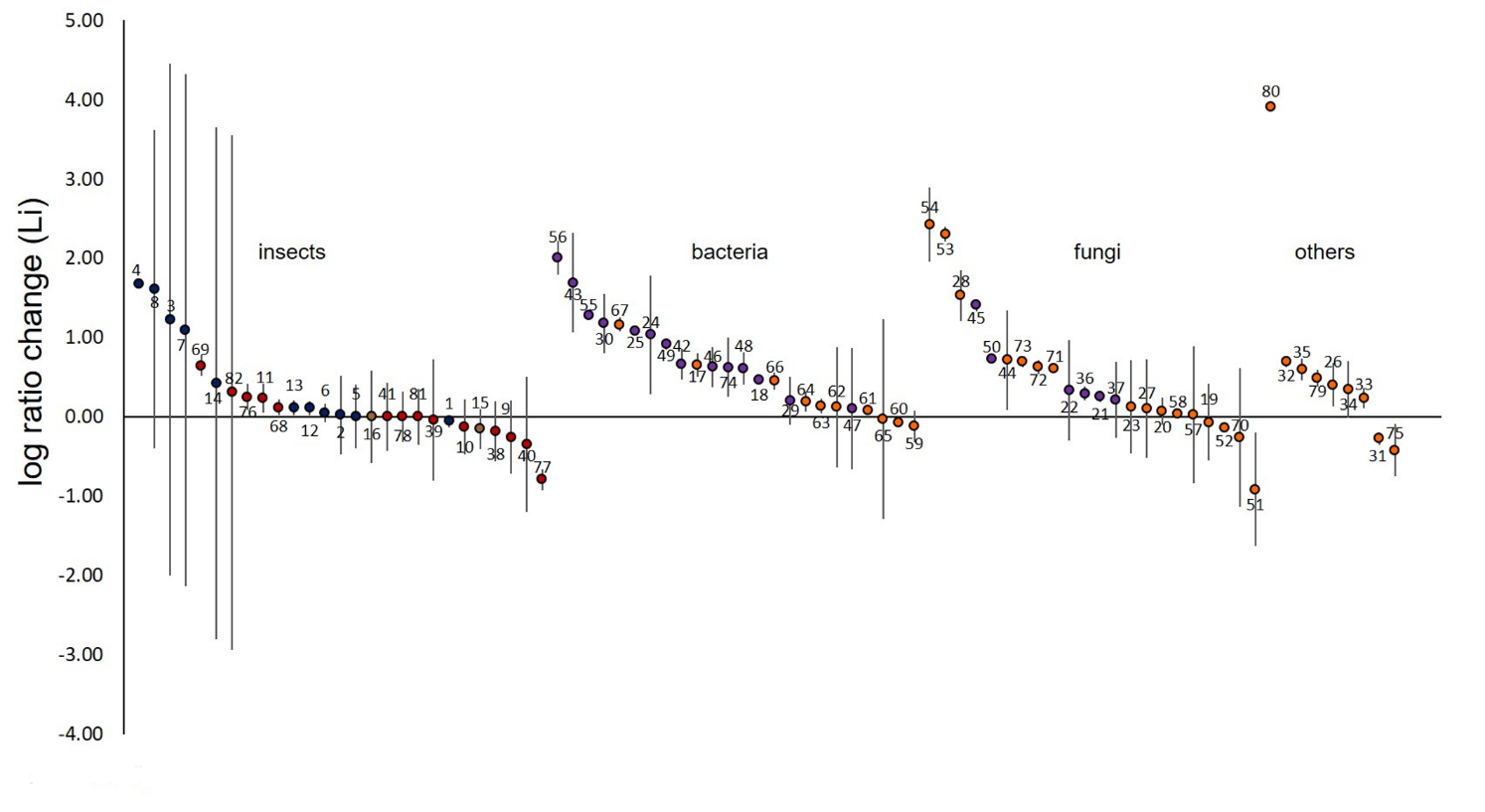
**

**Supplementary Figure 2.** Forest plot for log-ratio (L) representing change for flavonoids for each case separated by insect or pathogen type. For insects, red represents chewing insects, blue represents piercing-sucking insects, and brown represents wood-boring insects. For microbes, purple represents biological control agents and orange represents pathogens. Numbers above each point correspond to case number listed in Supplementary Table 2. Bars represent 95% confidence intervals.

**
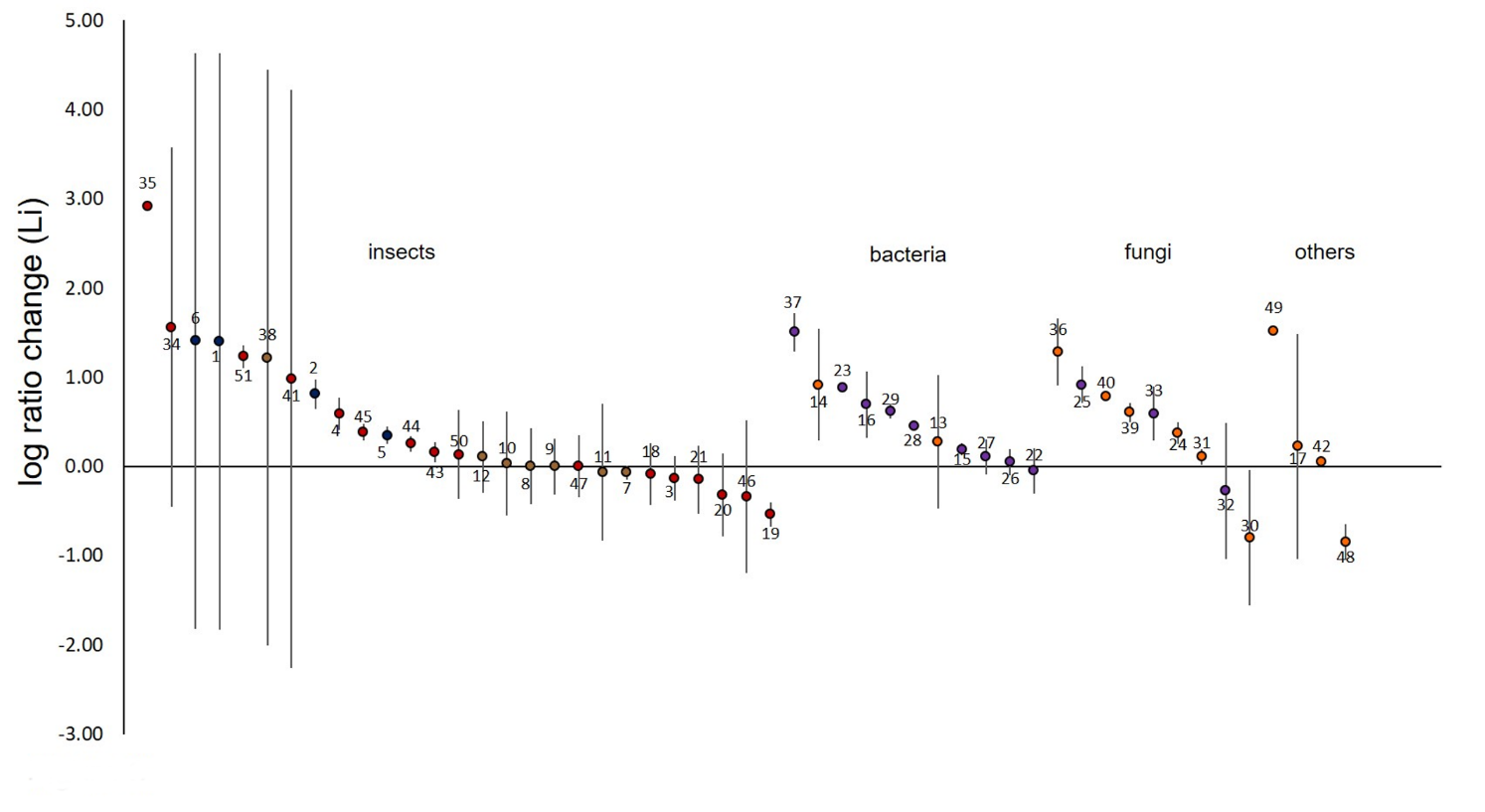
**

**Supplementary Figure 3.** Forest plot for log-ratio (L) representing change for hydroxycinnamic acid derivatives for each case separated by insect or pathogen type. For insects, red represents chewing insects, blue represents piercing-sucking insects, and brown represents wood-boring insects. For microbes, purple represents biological control agents and orange represents pathogens. Numbers above each point correspond to case number listed in Supplementary Table 3. Bars represent 95% confidence intervals.

**
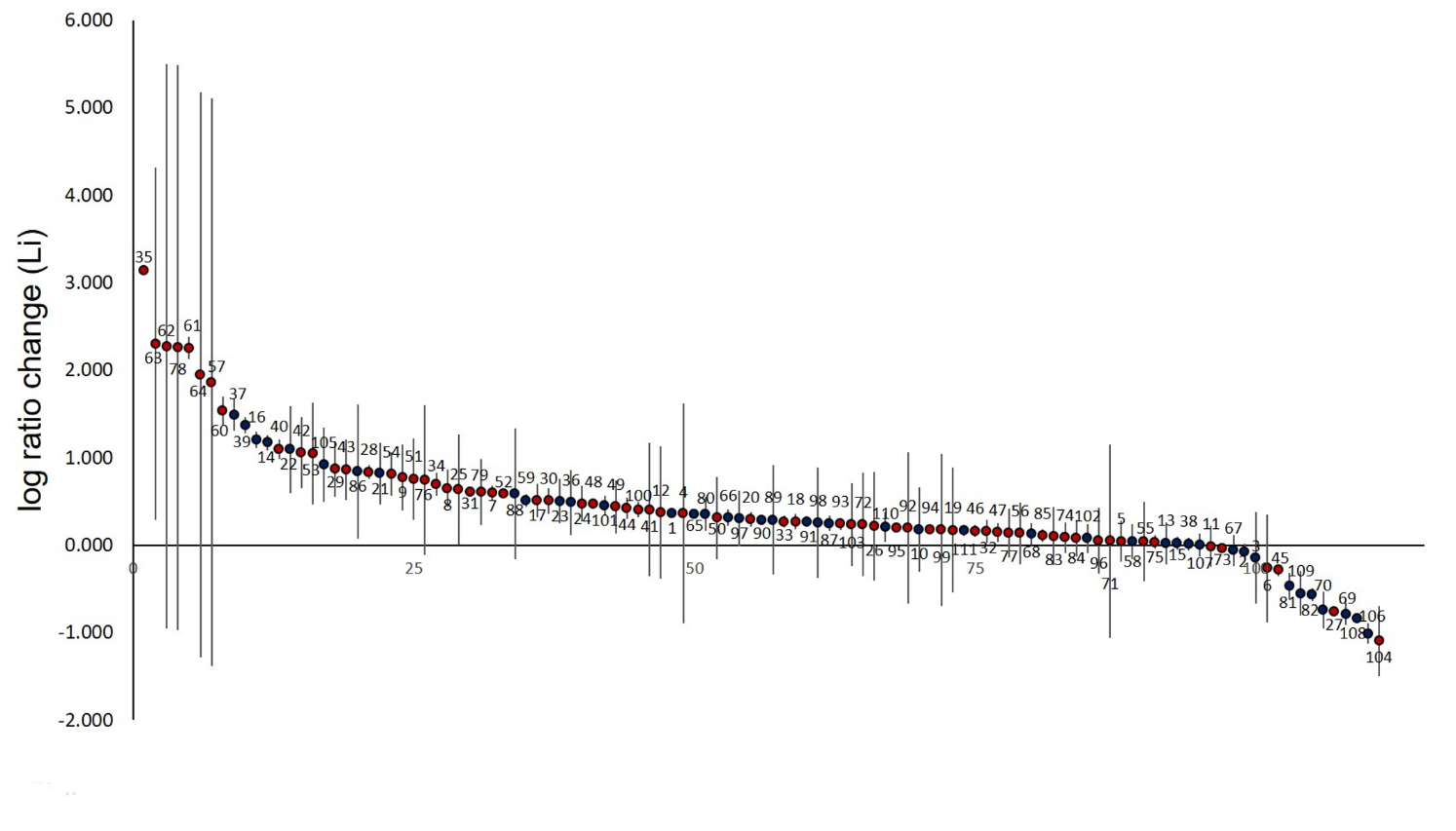
**

**Supplementary Figure 4.** Forest plot for log-ratio (L) representing change for total phenolics for each case separated by host type, with red representing annuals and blue representing perennial plants. Numbers above each point correspond to case number listed in Supplementary Table 1. Bars represent 95% confidence intervals.

**
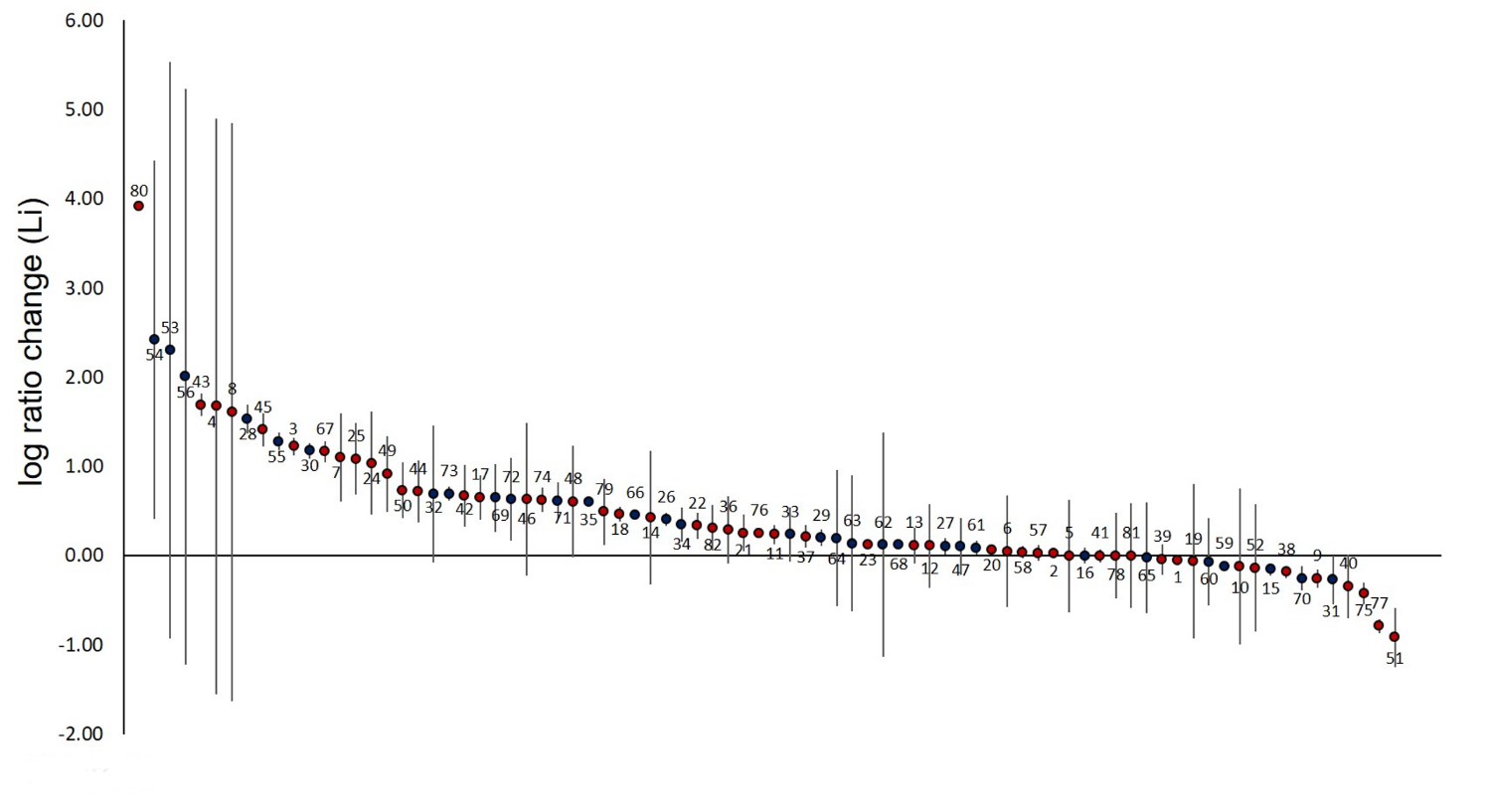
**

**Supplementary Figure 5.** Forest plot for log-ratio (L) representing change for flavonoids for each case separated by host type, with red representing annuals and blue representing perennial plants. Numbers above each point correspond to case number listed in Supplementary Table 2. Bars represent 95% confidence intervals.

**
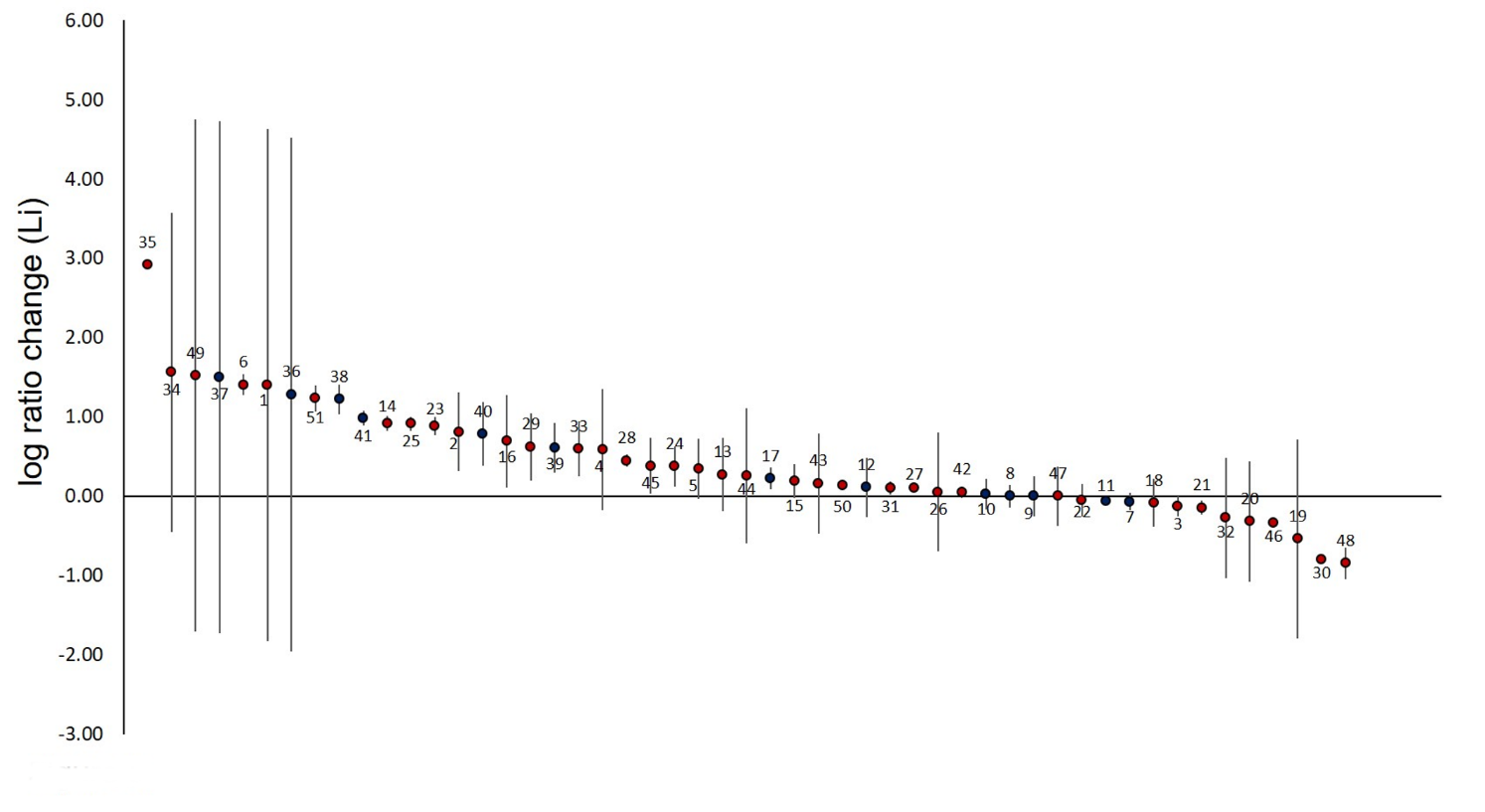
**

**Supplementary Figure 6.** Forest plot for log-ratio (L) representing change for hydroxycinnamic acid derivatives for each case separated by host type, with red representing annuals and blue representing perennial plants. Numbers above each point correspond to case number listed in Supplementary Table 3. Bars represent 95% confidence intervals.

**
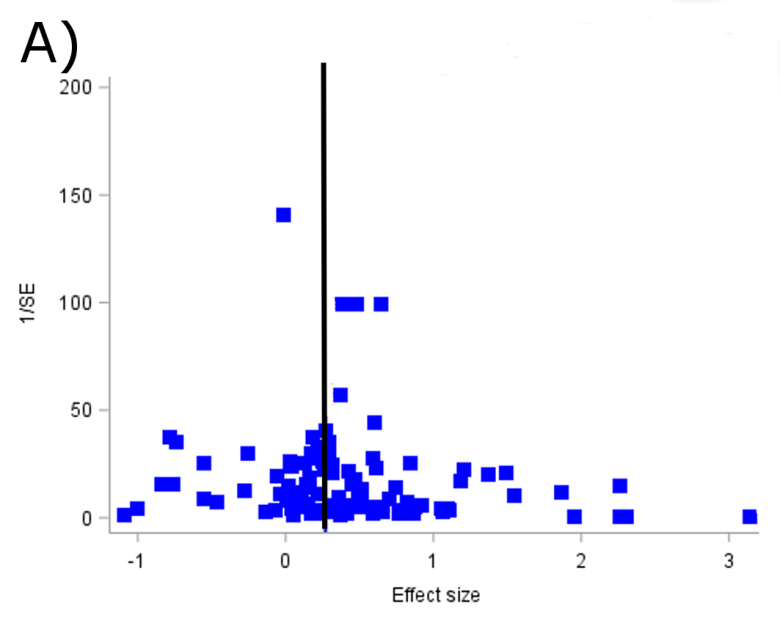

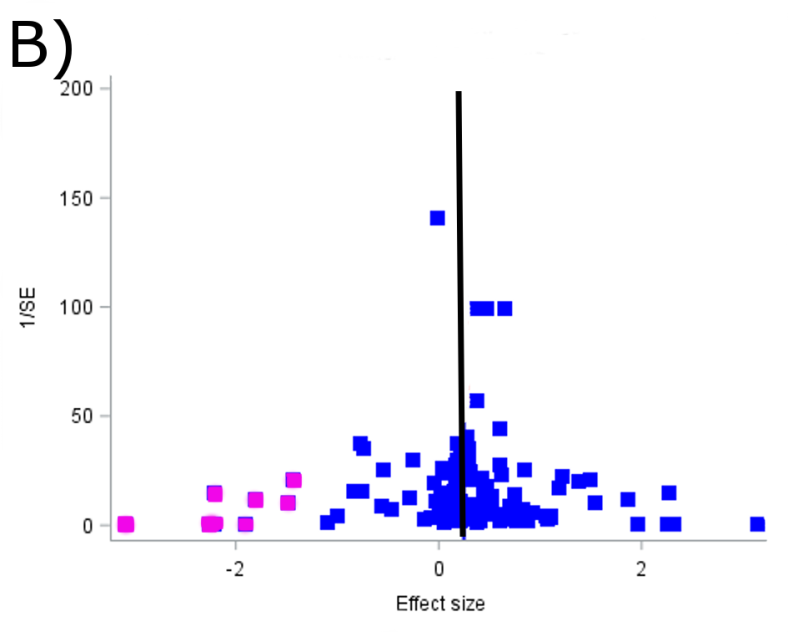
**

**Supplementary Figure 7.** A) Funnel plot for the total phenolics metadata used in this study, with the blue line with red dashes in the middle representing of the log ratio response mean. B) The funnel plot of the dataset adjusted via the “trim and fill” method, with additional data added to the left of the log ratio response mean to account for possible missing studies indicated in purple. Conclusions using the dataset from “trim and fill” were similar to the unadjusted dataset.


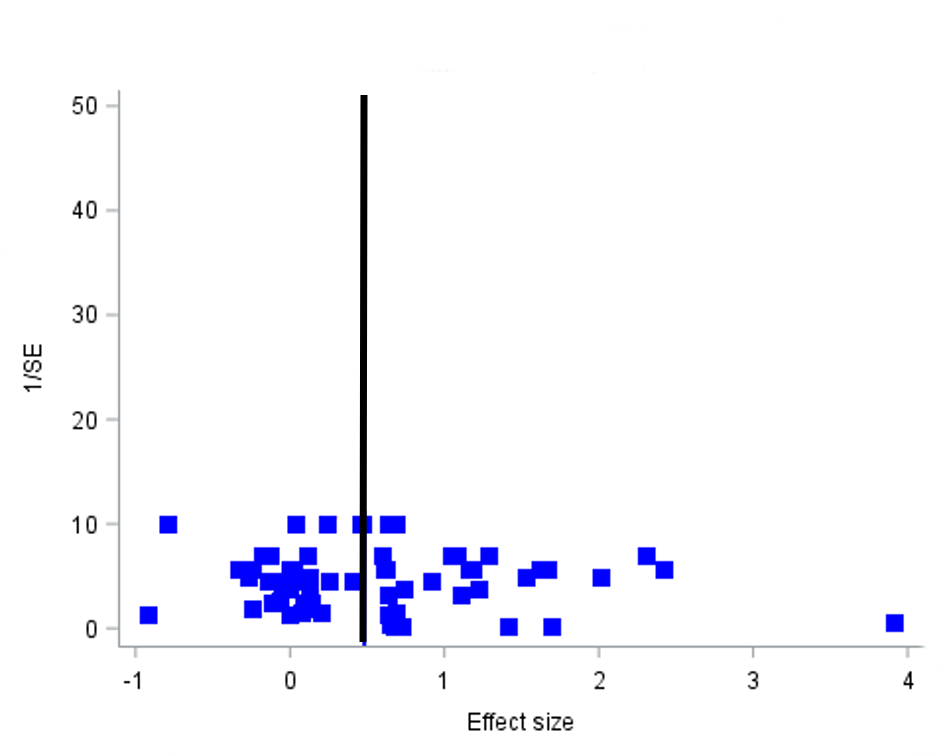


**Supplementary Figure 8.** Funnel plot for the flavonoid metadata used in this study, with the blue line with red dashes in the middle representing of the log ratio response mean.

**
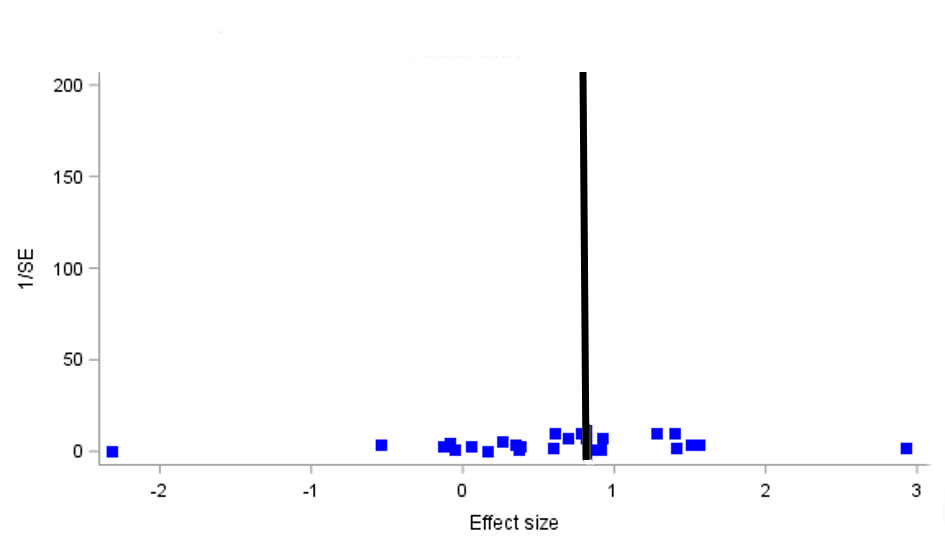
**

**Supplementary Figure 9.** Funnel plot for the hydroxycinnamic acid derivative (HCA) metadata used in this study, with the black line in the middle representing of the log ratio response mean.

**
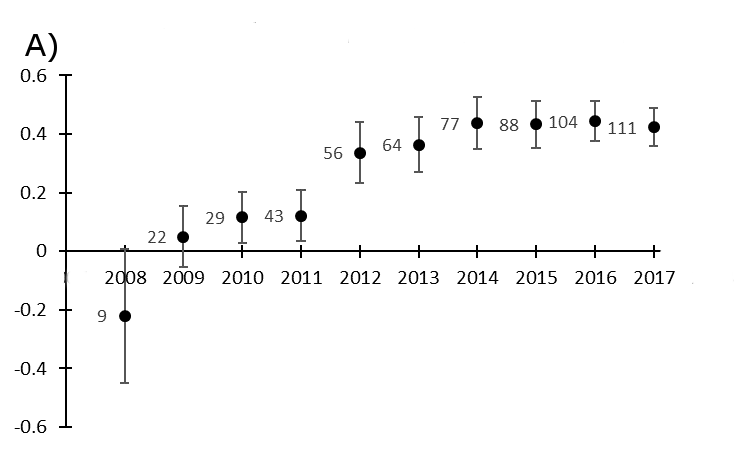

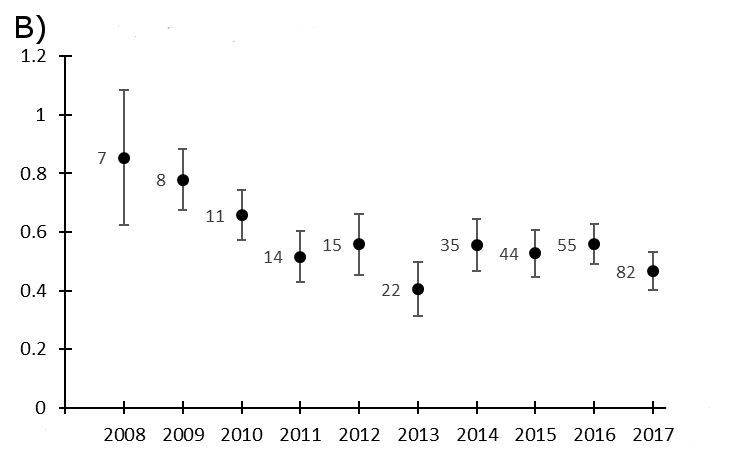

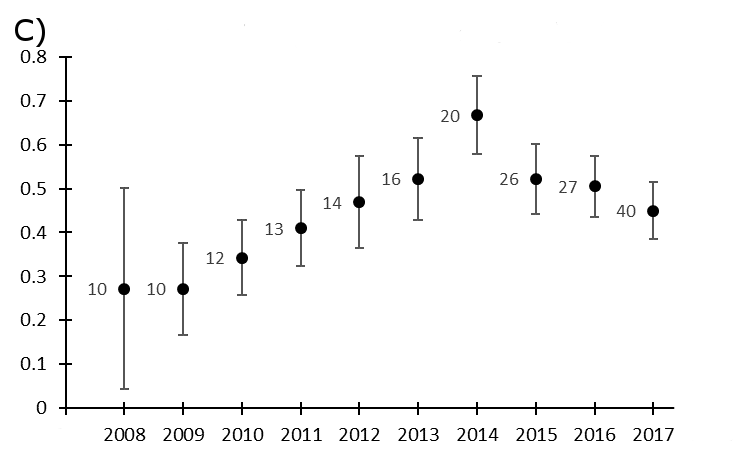
**

**Supplementary Figure 10.** Graphs showing temporal trends in cumulative grand mean effects sizes for A) total phenolics, B) flavonoids, and C) hydroxycinnamic acids. Cumulative grand mean effect sizes stabilized around 2012 for total phenolics, around 2011 for flavonoids, and 2013 for HCAs (with 2014 a possible outlier). Letters represent the cumulative number of case studies. Bars represent standard errors.

**
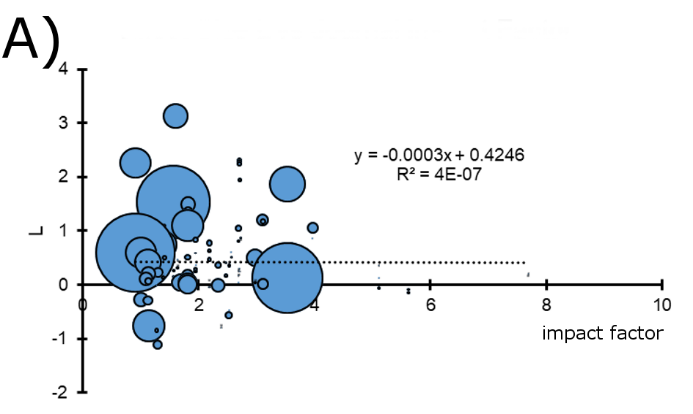

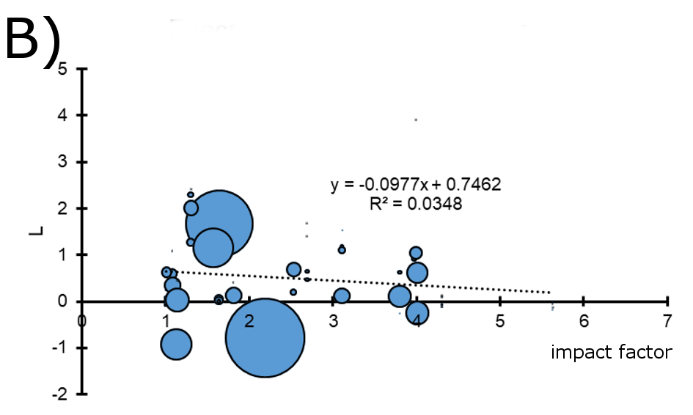

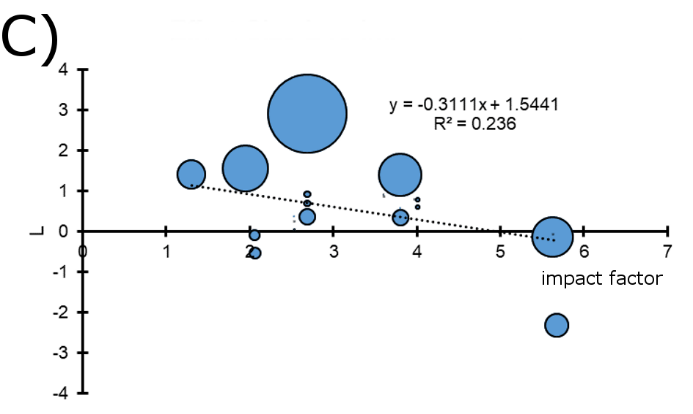
**

**Supplementary Figure 11.** Plots of study effect size (as log ratio response, L) versus publication journal impact factor for A) total phenolics, B) flavonoids, and C) hydroxycinnamic acid derivatives. Bubble size indicates the influence statistic of an individual value (Cook’s distance). Regression lines with R^2^ values are provided.
